# A Multi-Spectroscopic and Molecular Docking Analysis of the Biophysical Interaction between Food Polyphenols, Urolithins, and Human Serum Albumin

**DOI:** 10.3390/molecules29184474

**Published:** 2024-09-20

**Authors:** Nevena Zelenović, Predrag Ristić, Natalija Polović, Tamara Todorović, Milica Kojadinović, Milica Popović

**Affiliations:** 1 Institute of Chemistry, Technology, and Metallurgy, National Institute of the Republic of Serbia, University of Belgrade, Njegoševa 12, 11000 Belgrade, Serbia; nevenazelenovic@gmail.com; 2 Faculty of Chemistry, University of Belgrade, Studentski trg 12-16, 11000 Belgrade, Serbia; predrag@chem.bg.ac.rs (P.R.); polovicn@chem.bg.ac.rs (N.P.); tamarat@chem.bg.ac.rs (T.T.); 3 Institute of Medical Research, National Institute of the Republic of Serbia, University of Belgrade, Tadeusa Košćuška 1, 11000 Belgrade, Serbia; milica.kojadinovic.imr@gmail.com

**Keywords:** urolithins, human serum albumin, binding affinity, fluorescence spectroscopy, Fourier transform infrared spectroscopy, molecular docking

## Abstract

Secondary polyphenol metabolites, urolithins (UROs), have anti-oxidative, anti-inflammatory, and antidiabetic properties. Therefore, their biological activity relies on blood transport via human serum albumin (HSA) and tissue distribution. The main goal we set was to investigate the interaction between HSA and different URO (URO A, URO B, URO C, URO D, and glucuronidated URO A and B) using a combination of multi-spectroscopic instrumental and in silico approaches. The fluorescence spectroscopy revealed that URO can quench the naturally occurring fluorescence of HSA in a concentration-dependent manner. The HSA fluorescence was quenched by both a static and dynamic mechanism. The results showed that free UROs bind to HSA with higher affinity than their conjugated forms. CD spectroscopy and FTIR revealed that the alpha-helical structure of HSA is preserved. The calculated Gibbs free energy change indicates that the URO–HSA complex forms spontaneously. There is a single binding site on the HSA surface. The molecular docking results indicated that unconjugated Uro binds to Sudlow I, while their conjugation affects this binding site, so in the conjugated form, they bind to the cleft. Docking experiments indicate that all UROs are capable of binding to both thyroxine recognition sites of ligand-bound HSA proteins. Examining interactions under the following conditions (298 K, 303 K, and 310 K, pH 7.4) is of great importance for determining the pharmacokinetics of these bioactive compounds, as the obtained results can be used as a basis for modulating the potential dosing regimen.

## 1. Introduction

**Figure 1:**
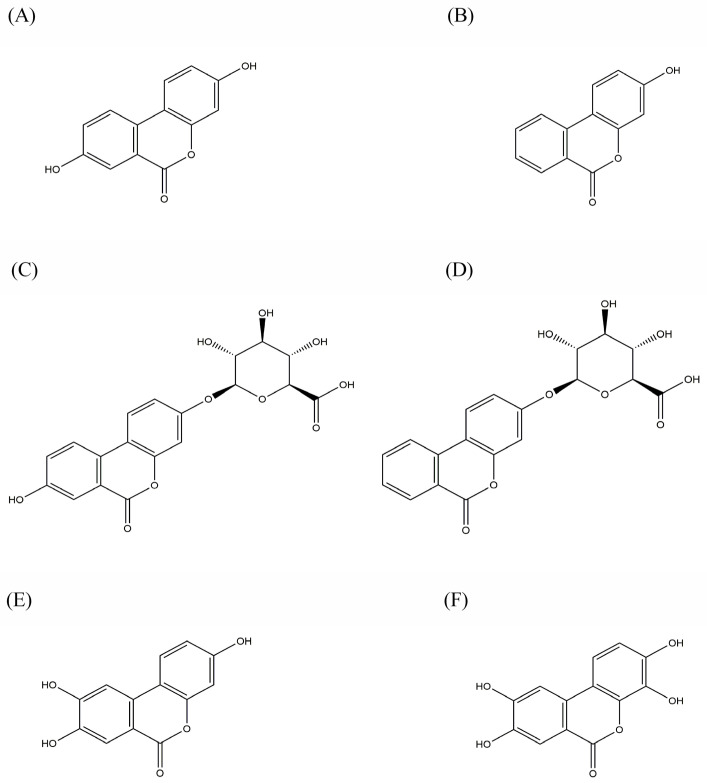
The chemical structures of urolithins are shown in the following order: (**A**) Urolithin A (URO A); (**B**) Urolithin B (URO B); (**C**) Urolithin A glucuronide (URO AG); (**D**) Urolithin B glucuronide (URO BG); (**E**) Urolithin C (URO C); (**F**) Urolithin D (URO D).

Polyphenol-rich foods have long been deemed beneficial sources of anti-inflammatory and anti-oxidative properties. Plant foods, including berries (raspberries, strawberries, and blackberries), tropical fruits (guavas and pomegranates), nuts (pecans and walnuts), teas (black and green tea), and oak-aged wines, contain non-flavonoid polyphenol components such as ellagitannins (ET) [1,2]. In the gut, due to the activity of intestinal microbes, ET undergoes hydrolysis to form ellagic acid (EA). EA has low intestinal absorption and undergoes the reaction of decarboxylation of the one lactone ring and subsequent elimination of hydroxyl groups, metabolizing to urolithins (URO). This process generates numerous URO isomers. Although UROs’ isomers have different numbers of hydroxyl groups, they are all derivatives of dibenzopyran-6-one. The catabolic pathway of EA to URO takes place from pentahydroxy-URO (URO M-5) via tetrahydroxy-URO (URO D, URO E, and URO M-6), trihydroxy-URO (URO C and URO M-7), and dihydroxy-URO (URO A and isoURO A) to monohydroxy-URO (URO B) [3]. Figure 1 displays the chemical structure of the ellagic acid metabolites used in this work.

Upon consumption of ellagitannin-rich foods, free and conjugated URO can be detected in human fluids (blood, urine, feces), as well as tissue (prostate, colon, and breast) [3]. In circulation, conjugated URO is found in the form of sulfates and glucuronides, which represent a more abundant form with concentrations ranging from 0.2 × 10^−6^ mol L^−1^ to 20.0 × 10^−6^ mol L^−1^ [4]. However, they show reduced biological activity compared to unconjugated isoforms [5]. Conjugated UROs are transported to tissues via circulation [6]. When delivered to the tissue, UROs deconjugate and exert their biological activity (anti-inflammatory, anti-carcinogenic, anti-glycative, antioxidant, and antimicrobial) [7]. Additionally, URO A, the primary metabolite, stimulates the autophagy of old and damaged mitochondria, as well as their replenishment inside cells, thereby maintaining mitochondrial biogenesis [8]. This property of URO A has been exploited for supplement production. The U.S. Food and Drug Administration (FDA) has approved the URO A-based Mitopure supplement, which enhances skeletal muscular endurance and delays cell aging, for use in humans [9,10].

Human blood plasma predominantly contains human serum albumin (HSA). HSA is a single polypeptide chain protein of 585 amino acid residues, containing 17 pairs of disulfide bridges and a free Cys residue that maintains its structure and heart-like shape [11]. It consists of three similar α-helical domains arranged into two subdomains: A and B. Hormones, fatty acids, and drugs that bind to the hydrophobic cavities of subdomain IIA (site I), subdomain IIIA (site II), and subdomain IB (site III) are moved and stored by this protein [12]. Sudlow sites I and II are the two most significant binding sites on the HSA molecule. Sudlow site I, located in subdomain IIA, binds heterocyclic compounds, such as warfarin. Sudlow site II, located on subdomain IIIA, binds aromatic compounds such as ibuprofen [13]. Internal fluorescence of HSA originates from one Trp214 residue and two Tyr150 and Tyr411 residues [11]. Site number I contains both, while site number II contains only Tyr residue. The maximum number of ligands that may bind to albumin is determined by the overall quantity of ligands in circulation as well as by their affinity and number of binding sites. Thus, localization and determination of the number of binding sites in the HSA are crucial for understanding the drug’s pharmacokinetics [13].

It is well known that phenolic compounds, such as URO, bind to HSA and quench part of its fluorescence. Fluorescence quenching can be used for measuring binding affinities. URO can bind bovine serum albumin (BSA), as demonstrated by the quenching of the intrinsic Trp214 fluorescence, and the binding affinity is affected by the hydrophobicity of the URO itself [14]. Examining the interactions of urolithins and HSA under simulated physiological conditions (pH 7.4) is critical for the pharmacokinetics of these bioactive compounds. Our goal was to study the biophysical and structural basis of the URO–HSA complexes by combining experimental (fluorescence quenching, synchronous fluorescence, Fourier transformed infrared spectroscopy, and CD spectroscopy) and computational methods (molecular docking). The obtained results can give us insight into the mechanism of molecular interactions between HSA and urolithins in vitro, which helps us understand the pharmacodynamics and transport of these bioactive compounds.

## 2. Results and Discussion

### 2.1. Urolithins Quenching the Intrinsic Fluorescence of HSA

**Figure 2:**
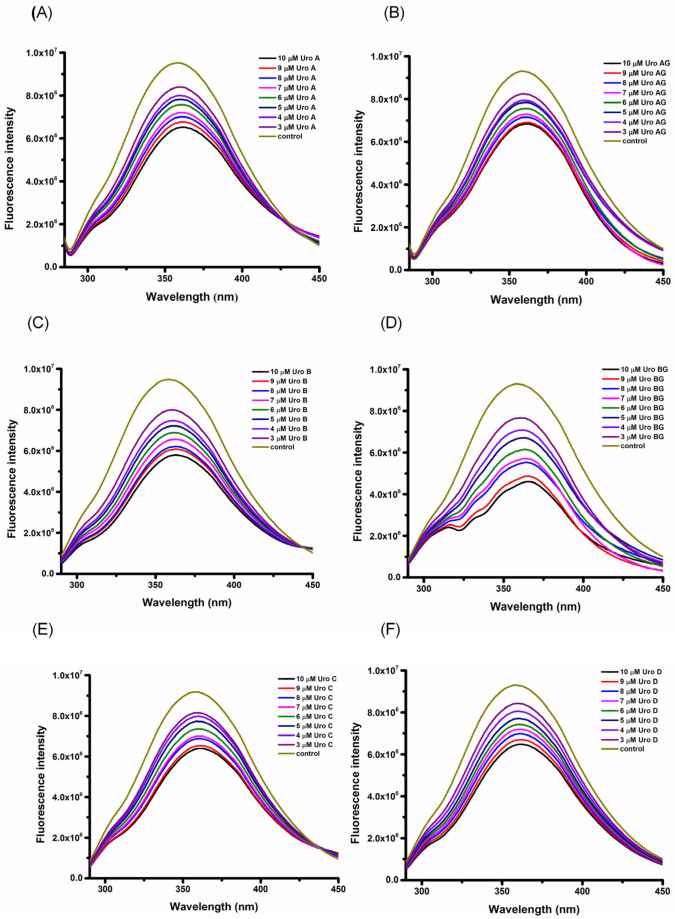
The fluorescence emission spectra of HSA in the presence of increasing concentrations of URO (**A**) URO A (**B**) URO AG (**C**) URO B (**D**) URO BG (**E**) URO C (**F**) URO D at excitation λ_ex_ = 280 nm. Conditions: pH = 7.4, T = 298 K. The HSA concentration was 3 × 10^−6^ mol L^−1^, whereas the URO concentration was increased from 3 × 10^−6^ mol L^−1^ to 10 × 10^−6^ mol L^−1^ at an increment of 1 × 10^−6^ mol L^−1^.

HSA has intrinsic fluorescence stemming from the presence of aromatic amino acid residues (Trp and Tyr), with a characteristic fluorescence peak at around 360 nm [15]. The HSA’s main intrinsic fluorophore is Trp214. The binding of phenolic compounds to HSA leads to subtle structural changes, consequently changing the microenvironment of the protein’s fluorophore and quenching the intrinsic HSA fluorescence [16]. To observe the quenching interactions of different URO with HSA, fluorescence emission spectra were recorded with an increasing amount of URO (3 × 10^−6^ mol L^−1^ to 10 × 10^−6^ mol L^−1^). Figure 2 displays the intrinsic fluorescence spectra of HSA at a temperature of 298 K and a pH of 7.4 in the presence of increasing concentrations of URO. When exited at a wavelength (λ_ex_) of 280 nm, the fluorescence emission spectrum of HSA displays characteristic peak at λ_em_ of 360 nm. The intrinsic fluorescence intensity of HSA decreases by approximately 30% after interacting with the highest concentration of URO (10 × 10^−6^ mol L^−1^) without a change in the absorption maximum λ_em_ or the shape of the peak. The quenching of HSA fluorescence is concentration-dependent, and a significant decrease in fluorescence intensity at λ_em_ can be observed (Figure 2). A similar trend in concentration-dependent fluorescence quenching can be observed at the other two temperatures, 303 K and 310 K (Supplementary Figures S1 and S2), with no observable changes in either absorption maximum λ_em_ or the shape of the peak. A lack of blue or red shift in fluorescence quenching during interactions between HSA and URO suggests that the ligand binding does not significantly alter the fluorophore’s local environment or the protein’s structure around the fluorophore [17].

### 2.2. Stern–Volmer Plots and Constants

**Figure 3:**
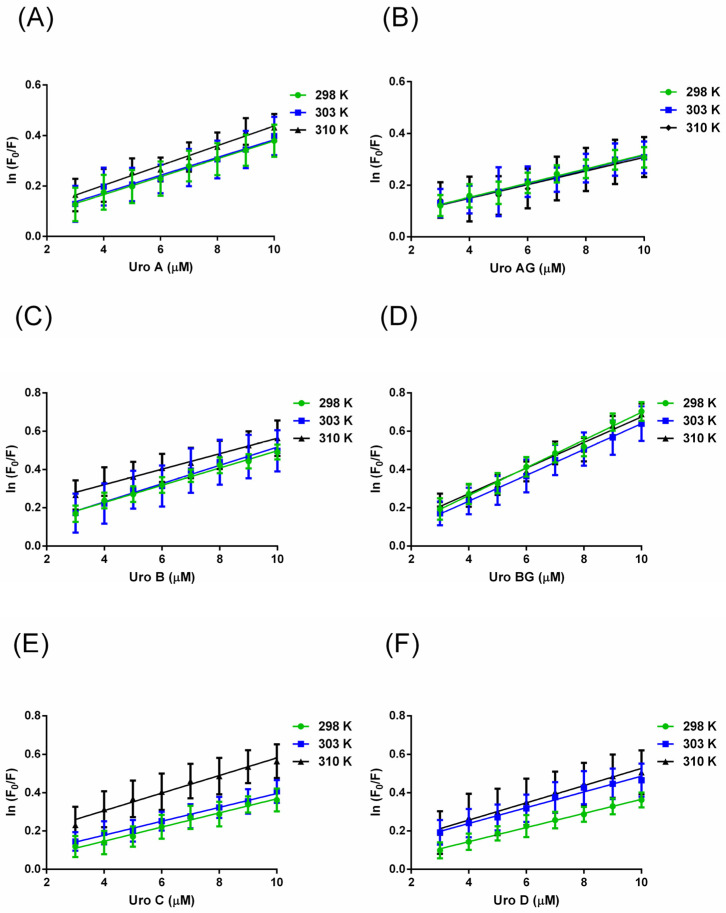
Modified Stern–Volmer plots were generated to analyze the quenching of HSA by several compounds, namely (**A**) URO A (**B**) URO AG (**C**) URO B (**D**) URO BG (**E**) URO C (**F**) URO D, at a temperature of 298 K (green), 303 K (blue), and 310 K (black), and a pH value of 7.4. Error bars indicate standard errors of triplicate measurements.

The fluorescence quenching of HSA can occur through static (the fluorophore and the quencher form a ground-state complex), dynamic (molecules collide in the transition to the excited state rather than direct interaction between the fluorophore and the quencher), or mixed (a combination of static and dynamic quenching) [18]. Stern–Volmer plots were constructed according to Equation (3). The relative fluorescence intensity data (F_0_/F) as a function of quencher concentration [Q] at 298 K, 303 K, and 310 K show linearity for the used range of concentrations, indicative of a static mechanism of quenching (Supplementary Figure S3). However, there is a pronounced change in the curve slopes with an increase in temperature, suggesting that there is a “sphere of action model” or that HSA could be quenched by both a static and dynamic process. According to this model, there is a volume sphere surrounding the fluorophore within which a quencher will cause quenching with a probability of unity. When the quencher is in close proximity to the protein’s fluorescence during the excitation process, quenching takes place. No ground state complex forms in this model. When there were both static and dynamic mechanisms present, the quenching data were analyzed using a modified version of the Stern–Volmer plot according to Equation (4). The values of K_sv_ were determined from the slope of the linear relationship between ln (F_0_/F) and [Q] (Figure 3). Furthermore, the obtained values for bimolecular quenching rate constants (k_q_) (Table 1) far exceed the upper limit for a dynamic quenching mechanism of ~10^10^ M^−1^·s^−1^ at 298 K, 303 K, and 310 K, additionally corroborating a sphere of action model [19].

### 2.3. Binding Constants and Number of Binding Sites

**Figure 4:**
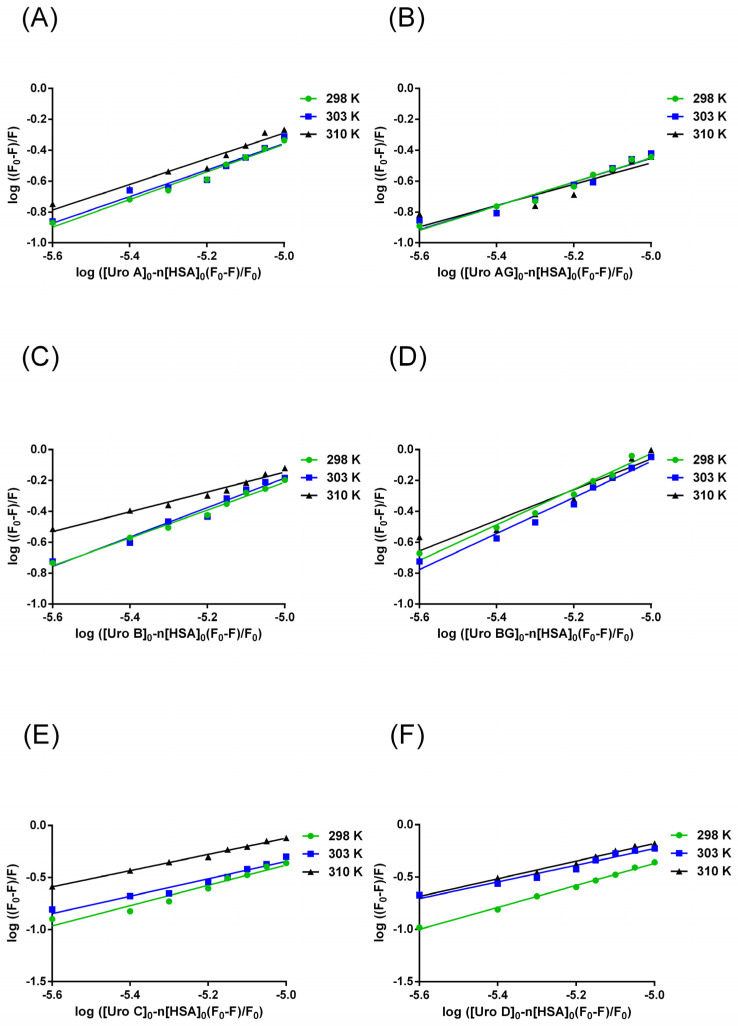
Double-log plots for the determination of binding constants, K_b,_ and number of binding sites n for (**A**) URO A (**B**) URO AG (**C**) URO B (**D**) URO BG (**E**) URO C (**F**) URO D to HSA (3 × 10^−6^ mol L^−1^) at 298 K, 303 K, and 310 K, and pH 7.4.

Using the double-logarithmic plot of the interaction of URO with HSA (Equation (6)), we obtained information on binding constants (K_b_) and the number of binding sites (n). The calculated value n for all URO is approximately 1, suggesting the presence of one binding site. The K_b_ values for URO–HSA complexes are in the range of 10^4^–10^5^ L mol^−1^, indicating a moderate binding affinity (Figure 4). Additionally, Kb decreases with increasing temperature, showing that the binding is better performed at a lower temperature (Table 1).

### 2.4. Thermodynamic Parameter and Interaction Modes

The change in Gibbs free energy (ΔG) for binding of each URO at different temperatures can be calculated from Equation (10). The obtained values for ΔG (Table 2) are negative, indicating that the reaction of URO–HSA complex formation is exergonic and spontaneous. Non-covalent interactions that form upon binding of a ligand to HSA include hydrogen bonds, salt bridges, hydrophobic and van der Waals interactions, π–π interactions, and steric contacts, and these define the strength of these interactions [20].

**Figure 5:**
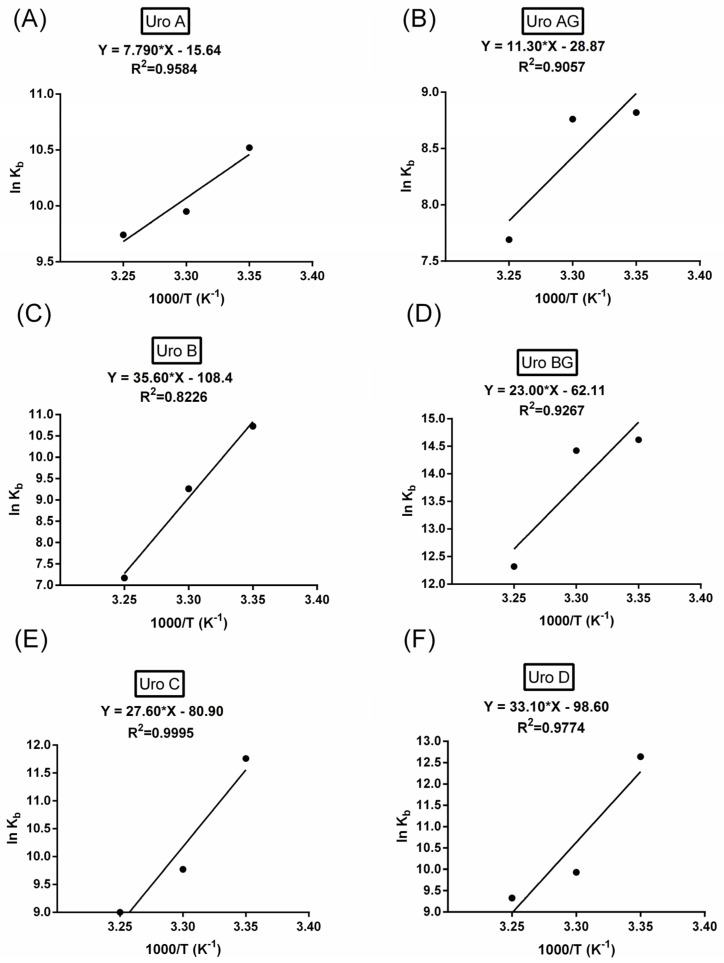
van’t Hoff plot for the interaction of HSA with (**A**) URO A (**B**) URO AG (**C**) URo B (**D**) URO BG (**E**) URO C (**F**) URO D.

The thermodynamic parameters, including the enthalpy change (ΔH) and entropy change (ΔS) during the process, are important for the study of the interaction force between HSA and URO. ΔH and ΔS can also be calculated from the van’t Hoff plot, where ΔH can be obtained from the slope, while the entropy change (ΔS) can be obtained from the intercept of the van’t Hoff plots (Figure 5). These parameters are shown in Table 2.

From the point of view of thermodynamics, the values of ΔH < 0 and ΔS < 0 indicate that the van der Waals interactions or hydrogen bond formation between HSA and urolithins is the reason for the formation of interactions [21]. All the URO show a decrease in Kb with increasing temperature, while ΔH is negative. This signifies that the decrease in entropy is dominant. The binding process becomes less favorable at higher temperatures due to the disadvantageous increase in the (−TΔS) term, which outweighs the benefits of the exothermic nature of the process. Thus, the interaction is driven more by enthalpic considerations, and the decrease in entropy leads to less favorable binding conditions as the temperature rises, as seen from the change in ΔG [22].

### 2.5. Synchronous Fluorescence Spectra of URO–HSA System

**Figure 6:**
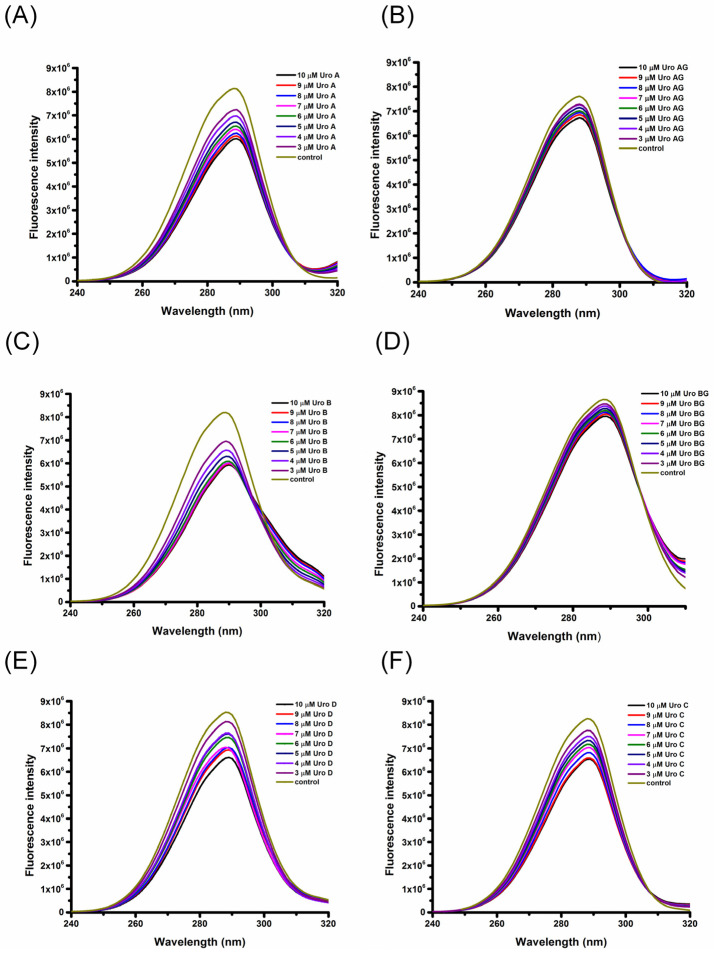
The impact of addition of increasing URO concentration on the synchronous fluorescence spectra of HSA at Δλ = 60 nm: (**A**) URO A; (**B**) URO AG; (**C**) URO B; (**D**) URO BG; (**E**) URO C; (**F**) URO D. The HSA concentration was 3 × 10^−6^ mol L^−1^, while the URO concentrations ranged 3 × 10^−6^–10 × 10^−6^ mol L^−1^ from top to bottom.

The technique of synchronous fluorescence was used to analyze the alternation in the microenvironments of the Tyr and Trp residues of HSA caused by the interaction with URO [23]. Simultaneously, the emission and excitation monochromators were scanned at a constant wavelength interval, Δλ (Δλ = λ_em_ − λ_ex_). Tyr residues were found to have a spectrum characteristic at Δλ = 15 nm, while Trp residues were found to have a spectrum characteristic at Δλ = 60 nm [24]. Tyr and Trp, two examples of aromatic amino acid residues, exhibit fluorescence emission peaks that are influenced by the polarity of their surroundings. The impact of URO addition on the synchronous fluorescence spectra of HSA at Δλ = 15 (Supplementary Figure S4) and Δλ = 60 nm wavelengths is displayed in Figure 6.

The obtained results demonstrated the presence of a single binding site on HSA for urolithins. Synchronous fluorescence shows us in which microenvironment changes occur. URO quenched fluorescence around both amino acid residues (Tyr and Trp) simultaneously. With increasing concentrations of URO, there is a decrease in fluorescence intensity around both residues. These data indicated that both amino acid residues participate in URO binding. Based on this, it can be concluded that site I is the primary location for the binding of aglycones (Figure 6 and Supplementary Figure S4). The decrease in fluorescence intensity of both residues for URO C and URO D (Figure 6 and Supplementary Figure S4) is somewhat less pronounced, indicating a weaker interaction with HSA at the Sudlow I site compared to URO A and URO B. The larger and more complex structures, as well as the presence of OH groups on a URO C and URO D, can affect its binding to the Sudlow I site of HSA by decreasing binding affinity by introducing steric hindrance, disrupting electrostatic interactions, or causing unfavorable conformational changes. For URO AG and URO BG, the Tyr and Trp residues’ maximum emission wavelengths did not change significantly, indicating that the binding of the glucuronidated URO occurs on different binding sites of HSA (Figure 6 and Supplementary Figure S4).

### 2.6. FT-IR Spectroscopy

The nature of the structural alterations the HSA underwent when it interacted with URO was demonstrated by the data obtained from FT-IR spectroscopy studies. Proteins’ peptide bond conformation can be analyzed within the Amide I, II, and III peaks. Proteins’ infrared spectra located in the amide I peak location were found to be ≈1600–1700 cm^−1^ arising from C=O stretch, whereas the amide II band was ≈1548 cm^−1^ is derived from NH in-plane bending and CN stretching mode [25,26]. Amide I spectra of HSA show a characteristic maximum at around 1650 cm^−1^ attributed to the protein’s most prominent secondary structure—α-helix [27]. Signal in the Amide I region from the C=O stretching vibrations of peptide bonds can be affected by the presence of carboxylic function of the Uro, and Uros’ interference can be observed especially at the wavenumbers 1660–1700 cm^−1^, making the Amide I region unsuitable for further structural analysis (Supplementary Figure S5). The Amide II region was found to be conformationally far less sensitive (Supplementary Figure S6), which is in concordance with previously published data [27]. Thus, we have decided to use the Amide III region (in which NH bending and the CN stretching vibrations contribute in a conformational-dependent manner) to observe the changes in the secondary structure of HSA after binding to URO.

**Figure 7:**
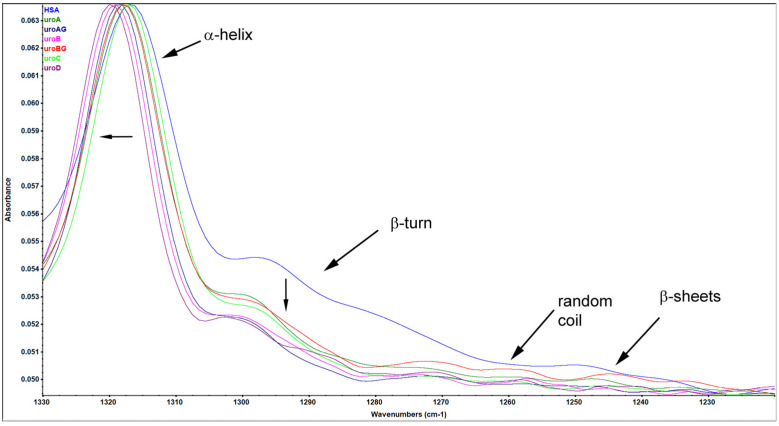
Amide III region of FTIR spectra of HSA in the absence and presence of URO (at pH 7.4).

Bands within the Amide III region were assigned to certain secondary structures as follows: 1330–1295 cm^−1^, α-helix; 1295–1270 cm^−1^, β-turns; 1270–1250 cm^−1^, random coils; 1250–1220 cm^−1^, β-sheets, as previously reported [28]. Figure 7 shows the Amide III region of the infrared spectra of HSA and URO–HSA samples. Spectra within amide III regions show preservation of the most dominant secondary structure, the α-helix, indicating that applied concentrations of URO did not cause massive structure loss in HSA. However, the most prominent changes upon URO binding are the slight red shift of the peak assigned to the α-helix and the lowered intensity of the β-turn peak, indicating predominant URO binding within protein regions containing these two structures (Figure 7).

### 2.7. Circular Dichroism (CD) Spectroscopy

CD spectroscopy is a simple, fast, and non-destructive technique that can provide structural information related to the asymmetry of molecules. Circular dichroism of peptide bonds is important for determining secondary structures of proteins because different secondary structures will have different intensities, positions of peaks, and shapes of bands in CD spectra. By monitoring secondary structures, the influence of ligand binding on protein conformation can be investigated [29,30].

**Figure 8:**
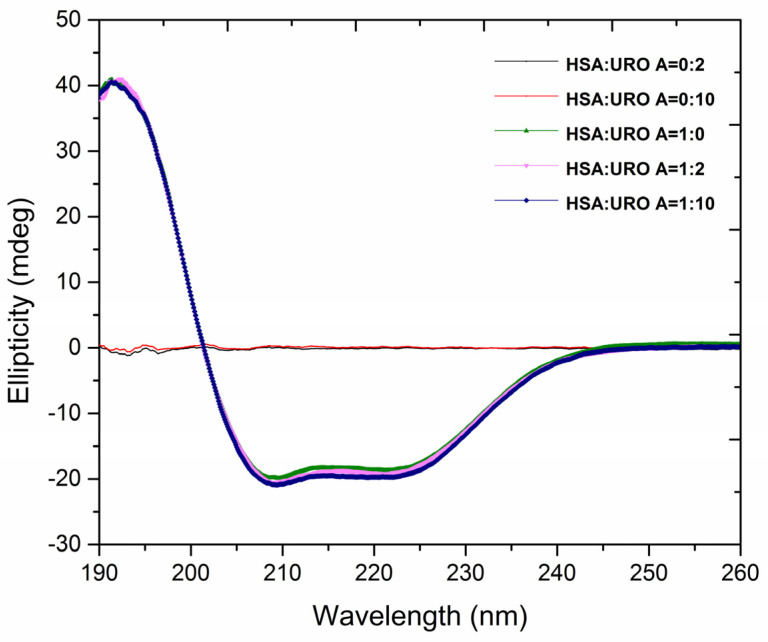
Far-UV CD spectra of free URO A (6.0 × 10^−6^ mol L^−1^; black line and 30.0 × 10^−6^ mol L^−1^; red line), free HSA (3.0 × 10^−6^ mol L^−1^; green line) and HSA-URO A complex (molar ratio 1:2; magenta line and 1:10; blue line) obtained at 298 K and pH 7.4.

The CD spectra of free HSA show two characteristic negative bands in the far-UV region at 208 nm and 222 nm (Figure 8), resulting from the α-helical protein structure [31]. The addition of URO A to the protein in a molar ratio of 2:1 and 10:1 did not lead to an increase in negative ellipticity, a shift in the peaks, or the shape of the spectrum of the protein (Figure 8). The CD spectrum of URO A was recorded in this region and did not show optical activity (Figure 8). The obtained spectra showed no changes in the secondary structures of HSA urolithin binding, indicating that the biding did not cause any observable change in the secondary structures of HSA, nor did it lead to destabilization of the protein and/or loss of signature helicoid arrangement.

### 2.8. Molecular Docking Studies

**Figure 9:**
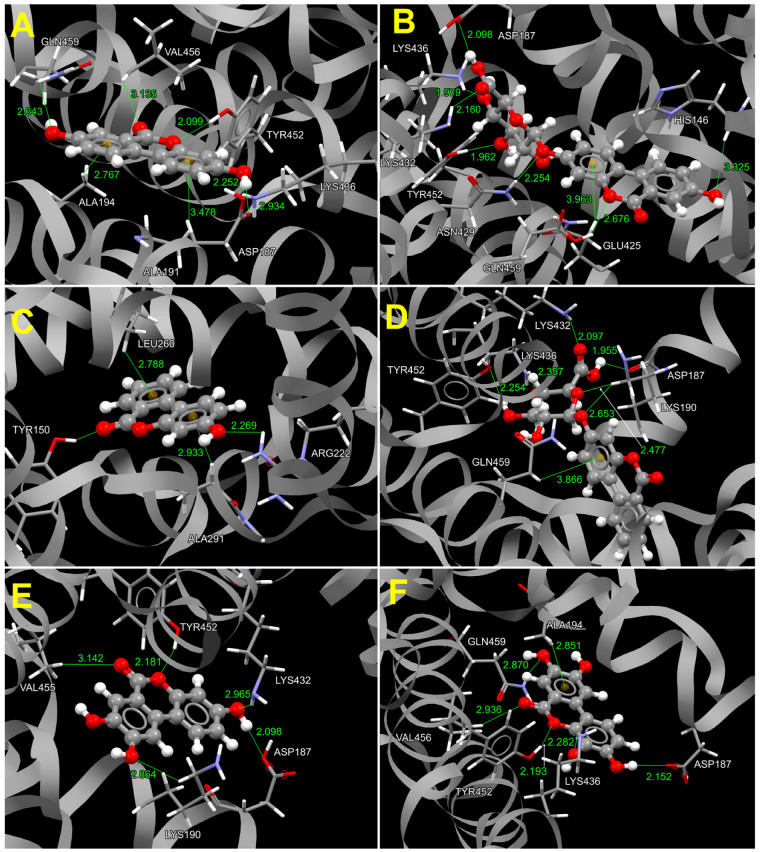
Three-dimensional bioactive conformations of URO A in FA9 (**A**), URO AG in FA9 (**B**), URO B in Sudlow’s site I (**C**), and URO BG in FA9 (**D**), URO C in FA9 (**E**), and URO D in FA9 (**F**), shown in ball and stick representation, with the corresponding molecular environment in the cavity. Amino acids that interact with ligands by non-covalent interactions (green lines) are shown in capped sticks style with a three-letter code and a sequence number in the protein sequence. The centroids of the aromatic rings are shown as ochre spheres. Amino acids and ligand atoms are represented by standardized CCDC colors (the gray color represents the urolithin carbon core, the red color represents the oxygen atoms, and the white color represents the hydrogen atoms from the hydroxyl group).

It is well known that HSA exhibits extraordinary ligand binding properties at multiple sites, where ligands can be various endogenous and exogenous low-molecular-weight compounds, as well as peptides and proteins [32]. A powerful experimental approach for investigating HSA–ligand interactions is fluorescence spectroscopy. However, experimental measurements can be further complemented with molecular docking studies that contribute to the understanding of structural chemistry and molecular recognition through the geometric analysis of protein–ligand interactions, as well as binding energies. Therefore, molecular docking studies of the investigated URO to ligand-free HSA were performed. The results of the molecular docking studies are given in Table 3, while Figure 9 shows the results of docking simulations of all six ligands. Schematic drawings of the interactions of the first GOLD cluster docked solutions for URO ligands, generated using LIGPLUS [33], are shown in Supplementary Figures S7 and S8. The observed preferences reflect the arrangement of hydrophilic/hydrophobic functional groups and the conformational freedom of the ligands.

The common feature of aglycones URO A and URO B is almost equal affinity for both binding sites, as seen from almost identical E_TOT_ and LE (Table 3). Both aglycones are positioned to make classical and/or non-classical hydrogen interactions with the hydroxyl group of Tyr150 in Sudlow’s site I, which is assumed to possess a central role in drug interactions [32]. In this binding site, URO A realizes classical hydrogen interactions with Arg257, Arg222, and Ala261, while hydrophobic interactions are established with residues Tyr150, His242, Leu238, Ala 291, Leu219, Leu269, and Ile290 (Supplementary Figure S7). Although the hydrophobic part of URO B is more dominant than in URO A, it also realizes a number of classical and non-classical hydrogen interactions in Sudlow’s site I (Figure 9C and Supplementary Figure S7). It acts as an acceptor in two C–H⋯π interactions with Leu260 and Ala 291 and one C–H⋯O interaction with Tyr150. URO B is a hydrogen donor in hydrogen interaction with Arg257 amide oxygen. With Tyr150, it achieved the classic O–H⋯O interaction, as well as N–H⋯O interaction with Arg222 residue, both acting as acceptors. Dominant interactions of URO A and URO B in cleft binding sites are depicted in 2D interaction plots (Supplementary Figure S8).

Due to the larger number of oxygen atoms in comparison to previous aglycones, URO C and URO D prefer binding to cleft binding sites (Table 3, Figure 9E,F). They realized a greater number of classical hydrogen interactions than the previous aglycones. URO C achieved hydrogen interaction with Tyr452, Lys436, Arg186, and Asp187. Residues involved in hydrophobic interactions with URO C are Lys432, Asn429, Ala194, Gln459, and Lys190. URO D also achieved a number of hydrogen and hydrophobic interactions.

Glucuronidation increases the hydrophilicity and conformational freedom of glycosylated URO. According to molecular docking, URO AG and URO BG bind preferentially in the cleft binding site (Table 3, Figure 9B,D, respectively), which is primarily constituted of polar and charged amino acids such as Tyr, Asn, Ser, Arg, and Lys. Docking scores indicate that the binding of aglycones to this binding site is significantly more favorable than affinity for the other binding site (Table 3). In the cleft binding site, URO AG achieves classical hydrogen interactions with Asp187, Lys190, Lys436, and Tyr452. URO BG realizes classical hydrogen interactions with Arg186, Asp187, Asn429, Tyr452, and Lys436. The binding in the cleft binding site is additionally stabilized by hydrophobic interactions with Lys190, Lys432, and Arg428 (Supplementary Figure S8).

Synchronous fluorescence spectroscopy revealed that Sudlow’s site I was the primary binding site for all non-conjugated urolithins, while both conjugated urolithins prefer the cleft pocket. The latter is corroborated by docking results. However, one should note that GOLD has been optimized for the prediction of probable ligand binding conformations rather than binding affinities.

**Figure 10:**
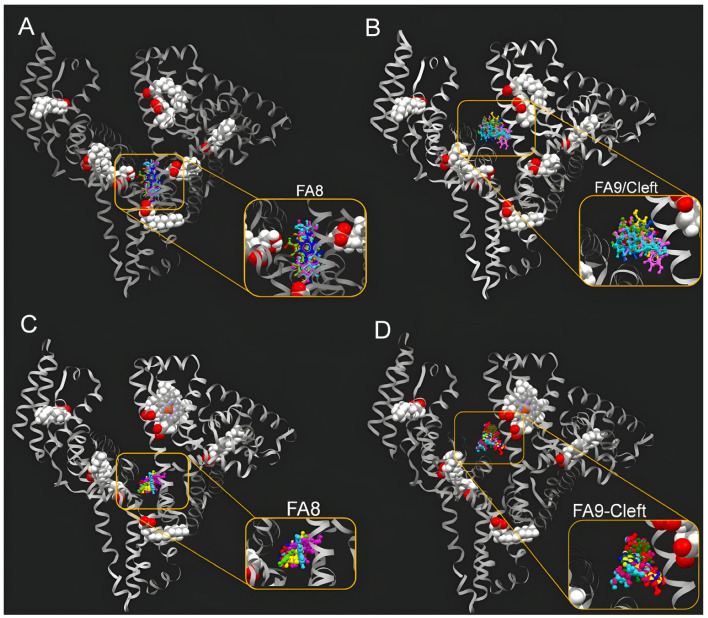
Superimposed 3D bioactive conformations of URO A (dark blue), URO AG (pink), URO B (yellow), URO BG (light blue), URO C (dark green), and URO D (red) in FA-HSA FA8 (**A**), FA-HSA FA9/Cleft (**B**), heme-HSA FA8 (**C**), and heme-HSA FA9/Cleft (**D**) binding pockets. Space-fill models were used to represent heme and myristic acid molecules, while urolithin ligand molecules were shown in ball and stick style.

In order to mimic the physiological conditions, the ligands were docked to binding pockets FA8 and FA9 of FA- and heme-HSA, which are relevant for thyroxine recognition. The results of the molecular docking study are given in Table 4, while Figure 10 depicts the results of docking simulations of all six ligands to both FA8 and FA9 sites. Schematic drawings of the interactions of the first GOLD cluster docked solutions for URO ligands, generated using LIGPLUS, are shown in Supplementary Figures S8–S12. Docking results showed that all URO are capable of binding to both thyroxine recognition sites of ligand-bound HSA proteins. However, further experimental studies are needed to validate the binding affinities of URO ligands.

## 3. Materials and Methods

### 3.1. Materials

The urolithins (URO A, URO AG, URO B, URO BG, URO C, and URO D) were generously supplied with a purity of 95% by Gonzalez-Sarrias. Some URO were synthesized based on the following procedure (URO A, URO B, and their conjugate URO AG and URO BG), and some URO (URO C and URO D) were purchased from Dalton Pharma Services (Toronto, Canada) [34]. Appropriately substituted benzoic acids (2-bromo-5-methoxybenzoic acid and 2-bromobenzoic acid) and resorcinol were used for the synthesis of URO A and URO B, respectively. The principle of synthesis is the condensation of these compounds under precisely defined conditions, during which a white powder precipitates. The resulting urolithins are then filtered and purified by HPLC. The identification of the purified URO is performed by confirming the molecular mass by the LC–MS system as well as by the ^1^H NMR spectrum [34,35].

The synthesis of URO B-glucuronide takes place in the presence of URO B as an acceptor of a glucuronosyl group and a donor of that group. The donor is a commercially available D-glucurono-6,3-lactone that has been derivative. The reaction takes place in the presence of the catalyst BF_3_-OEt_2_ and the solvent CH_2_Cl_2_. URO B is obtained in high yield (95%) under these conditions [34,36]. For the synthesis of URO A-glucuronides, URO A is first protected with silyl groups with a tert-butyldimethylsilyl (TBDMS) reagent. Protected URO A is the acceptor in the glycosylation reaction, which proceeds in the same way as the synthesis of URO B-glucuronide. After synthesis, ester hydrolysis and deprotection of acetyl and silyl groups is carried out using K_2_CO_3_ and KF in the methanol-water phase [34].

The HSA (fatty acid-free) was purchased from CSL Behring GMBH and used without any additional purification. The solution was prepared using water that had undergone a process of double distillation. All the other chemicals used in the experiment were of analytical grade.

### 3.2. Methods

#### 3.2.1. Measurement of Fluorescence Quenching of HSA

A stock solution (10.0 × 10^−6^ mol L^−1^) was prepared by diluting a HSA (MW 66, 500 Da) solution of 200 g/L in 0.1 mol L^−1^ phosphate buffer of pH 7.4 containing 0.15 mol L^−1^ NaCl and stored at 4 °C. Therefore, 3.0 × 10^−6^ mol L^−1^ HSA solutions were used for fluorescence quenching experiments and were titrated with a successive addition of URO. A total of 0.7 × 10^−6^ L of URO (in DMSO) was added to the protein solution to obtain URO solutions with concentrations in the range of 3.0 × 10^−6^ mol L^−1^ to 10.0 × 10^−6^ mol L^−1^. The prepared samples were excited at a wavelength of 280 nm. The fluorescence emission spectra were measured within the wavelength range of 200 to 600 nm at two temperatures (298 K and 310 K). All measurements of fluorescence were made against a blank solution. Appropriate blanks were subtracted to correct the background fluorescence. The influence of URO presence on the microenvironment of Trp214 residue of HSA was studied by measuring the fluorescence intensity using the spectrofluorometer FluoroMax-4 Model F-2000 (HORIBA Jobin Yvon, Kyoto, Japan) supplied with a 150 W ozone-free xenon arc lamp. A 1.00 cm quartz cell was used throughout the experiments. The widths of the slits used for excitation and emission were adjusted to 5.0 nm. All experiments were performed in triplicate.

The inner filter effect refers to a reduction in the measured intensity of fluorescence caused by the absorption of excitation light by the sample and subsequent emission of fluorescence [37]. For spectrofluorometric data to be accurate, this effect must be taken into account. The intensity of the measured fluorescence can be corrected according to the Lakowicz equation by measuring the absorption at the excitation and emission wavelengths [17] as follows:

(1) $$F_{corr}=F_{obs}*10^{\frac{A_{ex}+A_{em}}{2}},$$

after correction and observation, respectively. A_ex_ and A_em_ refer to the absorbance at the excitation and emission wavelengths, respectively. This effect can be neglected, and the correction of the measured fluorescence is not calculated when A is less than 0.07 at both wavelengths (A_ex_ and A_em_) [38,39].

A_ex_ and A_em_ were recorded on a BioTek Synergy LX multi-mode reader in a microtiter plate with a total volume of 300 × 10^−6^ L at corresponding λ (nm) and a temperature of 298 K. A total of 0.1 mol L^−1^phosphate buffer of pH 7.4 containing 0.15 mol L^−1^ NaCl was used as a blank solution. The A_ex_ and A_em_ of pure URO solutions (2.0 × 10^−6^ mol L^−1^–10.0 × 10^−6^ mol L^−1^) and HSA-URO spectra (2.0 × 10^−6^ mol L^−1^–10.0 × 10^−6^ mol L^−1^ URO and 3.0 × 10^−6^ mol L^−1^ HSA) were recorded. The obtained data for URO solution at corresponding concentrations were subtracted in the subsequent analysis [40].

#### 3.2.2. Stern–Volmer (SV) and Modified Stern–Volmer Equation

The fluorescence quenching data are analyzed using the Stern–Volmer equation that allows the determination of the binding affinity of fluorophore for quencher molecules and predicts the type of fluorescence quenching mechanism [16].

(2) $$\frac{F_{0}}{F}=1+{k_{q}\tau }_{0}\left[Q\right]=1+K_{SV}\left[Q\right]$$

where F_0_ represents the fluorescence emission intensity in the absence of a known concentration of quencher (URO), while F represents the fluorescence emission intensity in the presence of the known concentration of quencher (URO) [Q]. The resulting graph indicates the quenching mechanism. If a linear dependence of F_0_/F on the function Q is obtained, then one quenching mechanism is present. However, at high concentrations of Q, the SV plot exhibits an upward curvature, concave toward the *y*-axis. Then, the modified Stern–Volmer form of the equation is used:

(3) $$\frac{F_{0}}{F}=\left(1+K\left[Q\right]\right)\exp\left(\left[Q\right]VNa\right)$$

where V represents the volume in which the quencher is in contact with the chromophore, i.e., sphere-of-action volume, Na represents the Avogadro’s constant, and if K[Q] is a negligible value, then this equation takes the following form:

(4) $$\frac{F_{0}}{F}=e^{\left(K\left[Q\right]\right)}$$

Then, the dependence of ln(F_0_/F) as a function of [Q] is plotted, and a better linearity of the SV plot is obtained, from which the Stern–Volmer constant can be determined [41]. The Stern–Volmer quenching constant is represented by K_sv_. The quenching rate of the biomolecule is denoted by k_q_, and τ_0_ refers to the average fluorescence lifetime of HSA in the absence of the quencher, URO. The quenching rate constant, k_q_, is determined by employing the equation:

(5) $$k_{q}=\frac{K_{SV}}{\tau_{0}}$$

The k_q_ value was determined by utilizing the mean literature value of the fluorescence lifetime for HSA in the absence of any quenchers, denoted as τ_0_ = 10^−8^ s [19].

The double logarithmic equation

(6) $$\log\left(\frac{F_{0}-F}{F}\right)=\log Kb+nlog\left[Q\right]$$

was used to obtain values of the number of the binding site (n) and binding constant (K_b_) from the values of slope and y-intercepts, respectively [19].

In the case of the formation of a non-fluorescent complex between protein and ligand, the association constant (Kb) is calculated based on the following formula:

(7) $$\log\left(\frac{F_{0}-F}{F}\right)=\log Kb+nlog\left\{{\left[Q\right]}_{0}-n\frac{\left(F_{0}-F\right){\left[P\right]}_{0}}{F_{0}}\right\}$$

where ${\left[Q\right]}_{0}$ and ${\left[P\right]}_{0}$ represents the total concentration of quencher (URO) and total concentration of protein (HSA), respectively [42].

#### 3.2.3. Thermodynamic Parameters of URO–HSA Complexes

The following formula was used to calculate the Gibbs free energy change (ΔG) of the URO–HSA complex formation under the mentioned conditions (pH = 7.4; T = 298; 303 K and 310 K) [19].

(8) ΔG=−RTlnKb

Thermodynamic parameters, the enthalpy (ΔH), and entropy change (ΔS) during the process can be calculated based on the van’t Hoff Equation (9).

(9) $$lnKb=-\frac{\Delta H}{RT}+\frac{\Delta S}{R}$$

where K_b_ is the same as that of Equation (7), T is the absolute temperature, and the universal gas constant R is 8.314 JK^−1^ mol^−1^.

The Gibbs free energy change (ΔG) at different temperatures can be calculated from the following Formula (10) [42]:

(10) ΔG=ΔH−TΔS

#### 3.2.4. Measurement of Synchronous Fluorescence Spectrum

Synchronous fluorescence spectra were recorded with a FluoroMax-4 Model F-2000 (HORIBA Jobin Yvon, Kyoto, Japan) with a quartz cuvette with a route length of 1 cm and a volume of 700 × 10^−3^ L. In order to determine the sites where URO binds to HSA molecules, the synchronous fluorescence spectra of HSA solutions were recorded. This involved increasing the concentrations of URO and analyzing the spectra within the wavelength range of 240 nm to 400 nm. The range of synchronous scanning was λex = 240 nm to λem = 255 nm, where the difference in the wavelength (Δλ) was 15 nm, and λex = 240 nm to λem = 300 nm, where the difference in the wavelength (Δλ) was 60 nm.

#### 3.2.5. Fourier Transform Infrared Spectroscopy (FTIR)

HSA’s infrared spectra were obtained in ATR mode using a Nicolet Summit FTIR Spectrometer (Thermo Fisher Scientific, Waltham, MA, USA) with the addition of 30 × 10^−6^ mol L^−1^ URO. Tiny 1.5 × 10^−6^ L aliquots with a protein concentration of 1 mg/mL were placed on a diamond crystal, and the solvent was removed using an argon stream. Through 64 scans, composite spectra of the mid-IR (400–4000 cm^−1^) were obtained with the DTGS KBr detector. The background absorption was automatically adjusted in the spectra. Two fundamental additional corrections were made using OMNIC 7.0 software: baseline correction and automatic ATR correction.

#### 3.2.6. Circular Dichroism (CD) Analysis

CD spectra of HSA with or without ligand were recorded on a Jasco J-815 spectropolarimeter (JASCO, Tokyo, Japan) under the constant nitrogen flush at room temperature (298 K). Far-UV CD spectra of 3 × 10^−6^ mol L^−1^ HSA in the presence and absence of URO A were recorded in the range 190–260 nm, using a cell with a 0.5 mm path length, the scan speed of 50 nm min^−1^, and with an accumulation of three scans. The concentration of HSA was kept at 3.0 × 10^−6^ mol L^−1,^ and the concentrations of URO A were 6 × 10^−6^ and 30 × 10^−6^ mol L^−1^, respectively. The buffer blank background was subtracted from all CD spectra.

#### 3.2.7. Molecular Docking

The crystal structures of ligand-free HSA, myristic acid-bound HSA (FA-HSA), and heme-Fe(III)- and myristic acid-bound HSA (heme-HSA; PDB IDs: 1BM0, 8RCP, and 1N5U, respectively) [43,44,45] were extracted from the RCSB Protein Data Bank (www.rcsb.org) [46]. The three-dimensional (3D) structures of URO A–D, URO AG, and URO BG were built using ChemBio3D Ultra 12.0, followed by force field MM2 energy minimization [47]. Preparation of each protein structure (protonation, removal of water molecules, setting of atom and bond types, as well as adjustment of the flexibility of amino acid side chains and residues building up the walls of canonical HSA sites) was performed using the GOLD (Genetic Optimization for Ligand Docking) program implemented in the CSD-Enterprise Suite version 2022.3.0 [48,49,50,51]. With the intention of finding the bioactive conformation of the ligands, the conformational freedom of the ligand structures implied full torsion angle distribution and rotations, as well as the flipping of groups at the ring’s corners. Each ligand was docked to Sudlow’s I and Cleft binding sites of ligand-free HSA in the generated cavity of a 10 Å radius. For FA- and heme-HSA, the ligands were docked to FA8 and cleft binding pockets in the generated cavity of a 10 Å radius. CHEMPLP was chosen as a fitness function. The standard default settings were used in all calculations, and the ligands were submitted to 50 genetic algorithm runs. Results differing by less than 1.0 Å in ligand-all atom rmsd were clustered together. The best GOLD-calculated conformation was used both for analysis and representation. Distribution of productive poses by cluster analysis of docking results using a 1.0 Å RMSD is provided in Supplementary Material (Supplementary Figures S13–S15). Ligand efficiency (LE) was calculated according to the formula for LE = Etot/n, where *Etot* is the binding free energy (in kcal/mol) and n is the number of heavy atoms in the ligand. The number of heavy atoms for each compound was calculated from the molecular formula: URO A: 17 heavy atoms; URO B: 17 heavy atoms; URO AG: 20 heavy atoms; URO BG: 21 heavy atoms; URO C: 18 heavy atoms; URO D: 19 heavy atoms.

#### 3.2.8. Statistical Analysis

Statistical analysis was performed using Graph Pad Prism version 6. All measurements were conducted in triplicate, and the obtained data were expressed as the mean ± standard deviation in the fluorescence quenching analysis. K_sv_ values were calculated from linear regression.

## 4. Conclusions

Urolithins (UROs) do not naturally occur in the environment and must be obtained through ET-rich foods or dietary supplements. Once ingested, they enter the digestive tract and need to be transported through the bloodstream. Urolithins show considerable promise for their health benefits due to their potent antioxidant and anti-inflammatory properties. These compounds have been associated with a reduced risk of chronic diseases such as cardiovascular disease and cancer, thanks to their ability to neutralize oxidative stress and modulate inflammatory pathways [8]. Additionally, emerging research indicates that urolithins can positively influence gut health by affecting the microbiota, which may lead to improved metabolic functions and a lower incidence of metabolic syndrome [52]. Their ability to interact with cellular signaling pathways further underscores their potential as valuable components in functional foods and nutraceuticals aimed at enhancing overall health. Studying how UROs interact with HSA is crucial for gaining a deeper understanding of their pharmacological effects, metabolism, and transport within the circulatory system [53].

In summary, this study demonstrated the molecular binding of URO to HSA under simulated physiological conditions using multi-spectroscopic approaches and computational methods. The fluorescence spectroscopy experiments revealed that urolithins could bind to HSA and quench its intrinsic fluorescence. Considering the quenching rate constant (kq) values, which are on the order of 10^12^ L∙mol^−1^∙s^−1^, it may be concluded that the process of Trp fluorescence quenching is classified as a “sphere of action” model or a combination of static and dynamic quenching. The K_b_ values were in the order of 10^4^–10^5^ L·mol^−1^. The obtained n values were close to 1, indicating a single binding site on HSA for urolithins. The fact that Gibbs free energy change values are negative at all 298 K, 303 K, and 310 K shows that the formation process of the URO–HSA complexes is thermodynamically beneficial. All the URO show a decrease in Kb with increasing temperature, while ΔH is negative. This signifies that the binding process becomes less favorable at higher temperatures due to the disadvantageous increase in the (−TΔS) term, which outweighs the benefits of the exothermic nature of the process. Thus, the interaction is driven more by enthalpic considerations, and the decrease in entropy leads to less favorable binding conditions as the temperature rises, as seen from the change in ΔG. Glucuronidation contributes to the temperature sensitivity of protein–ligand binding, indicating that it further destabilizes interactions with HSA at higher temperatures. Similarly, glucuronidation affected the binding of URO to BSA, with aglycones displaying increased binding affinities [14].

URO binding to HSA did not seem to cause any significant change in the structure of HSA, as evident from both CD spectroscopy and FTIR. Spectra within amide III regions show preservation of the most dominant secondary structure, the α-helix, indicating that applied concentrations of URO did not cause massive structure loss in HSA. This was additionally confirmed using UROA in CD spectroscopy. Obtained spectra showed no changes in the secondary structures of HSA urolithin binding, indicating that the biding did not cause any observable change in the secondary structures of HSA, nor did it lead to destabilization of the protein and/or loss of signature helicoid arrangement.

Synchronous fluorescence and molecular docking were used to determine the URO binding site on HSA. Because URO quenches fluorescence around both Tyr and Trp residues concurrently in site I, synchronous fluorescence spectroscopy revealed that site I was the primary binding site for non-conjugated urolithins. However, docking of aglycones to ligand-free HSA predicted that URO C and URO D slightly prefer cleft binding sites, while for URO A and URO B, almost equal affinity for both sites was observed. For conjugated URO, synchronous fluorescence spectroscopy indicated little to no change in Tyr and Trp residues’ maximum emission wavelengths, while molecular docking suggested that the cleft binding site would be the primary binding location on HSA. Since GOLD has been optimized for the prediction of ligand binding conformations rather than binding affinities, further experimental studies, along with molecular dynamic simulations, were needed for confirmation of binding affinities of URO ligands. Examining interactions under the following conditions (298 K, 303 K, and 310 K, pH 7.4) is of great importance for the pharmacokinetics of these bioactive compounds. The obtained data could be used as a basis for modulating the potential dosing regimen of URO. In order to mimic the physiological conditions, the ligands were docked to binding pockets FA8 and FA9 of FA- and heme-HSA, which are relevant for thyroxine recognition. Docking results showed that all URO are capable of binding to both thyroxine recognition sites of ligand-bound HSA proteins.

The findings enhance our understanding of how free and conjugated urolithins bind to HSA, thereby broadening our knowledge of their pharmacodynamics and circulatory transport mechanisms. Furthermore, incorporating additional c techniques, such as isothermal titration calorimetry, nuclear magnetic resonance (NMR) spectroscopy, site displacement experiments, molecular dynamics, and site-directed mutagenesis of HSA amino acids, could provide deeper insights into the interactions between urolithins, urolithin glucuronides, and HSA.

## Figures and Tables

**Table 1:** The Stern–Volmer constants, quenching rate constants, and binding parameters for the interaction of different URO with HSA at 298 K, 303 K, and 310 K and pH 7.4.

Urolithin	pH	T (K)	K_sv_ (Lmol^−1^) × 10^4^	Kq (Lmol^−1^s^−1^) × 10^12^	^a^R^2^	S.D.	Kb (Lmol^−1^) × 10^4^	n	^b^R^2^
URO A	7.4	298	3.52	3.52	0.9959	0.0928	3.71	0.975	0.9927
		303	3.54	3.54	0.9767	0.2214	2.1	0.925	0.9608
		310	3.92	3.92	0.9878	0.1778	1.7	0.893	0.9817
URO AG	7.4	298	2.75	2.75	0.9883	0.1222	0.68	0.848	0.98
		303	2.73	2.73	0.9835	0.1442	0.64	0.842	0.9714
		310	2.64	2.64	0.9696	0.1997	0.22	0.758	0.9119
URO B	7.4	298	4.5	4.5	0.9922	0.163	4.59	0.961	0.993
		303	4.78	4.78	0.983	0.2564	1.05	1.026	0.9892
		310	4.83	4.83	0.9913	0.1543	0.13	0.774	0.9848
URO BG	7.4	298	7.24	7.24	0.9942	0.2708	222.84	1.253	0.9952
		303	6.74	6.74	0.9985	0.1181	183.23	1.286	0.9972
		310	6.67	6.67	0.9791	0.3103	22.49	1.064	0.952
URO C	7.4	298	3.62	3.62	0.9896	0.1547	12.85	1.085	0.9819
		303	3.69	3.69	0.9886	0.1514	1.76	0.907	0.9784
		310	4.59	4.59	0.9798	0.2692	0.81	0.794	0.988
URO D	7.4	298	3.69	3.69	0.9967	0.092	30.9	1.158	0.9947
		303	4.11	4.11	0.9827	0.2231	2.06	0.886	0.9892
		310	4.5	4.5	0.9854	0.2236	1.13	0.884	0.9876

^a^R is the correlation coefficient for the K_SV_ values. S.D. is the standard error of K_SV,_ and ^b^R is the correlation coefficient for the Kb values.

**Table 2:** The thermodynamic parameters of the URO–HSA complex at three different temperatures (298 K, 303 K, and 310 K) and pH 7.4.

Urolithin	pH	T (K)	ΔH (kJ mol^−1^)	ΔS (J mol^−1^K^−1^)	ΔG (kJ mol^−1^)	R^2^
URO A	7.4	298	−64.77	−130.03	−26.03	0.9584
		303			−25.38	
		310			−24.47	
URO AG	7.4	298	−93.95	−240.02	−22.42	0.9057
		303			−21.22	
		310			−19.54	
URO B	7.4	298	−295.98	−901.24	−27.41	0.8226
		303			−22.98	
		310			−16.59	
URO BG	7.4	298	−191.22	−516.38	−37.34	0.9267
		303			−34.76	
		310			−31.14	
URO C	7.4	298	−229.47	−672.6	−29.03	0.9995
		303			−25.67	
		310			−20.96	
URO D	7.4	298	−275.19	−819.76	−30.9	0.9774
		303			−26.8	
		310			−21.06	

**Table 3:** CHEMPLP total binding energies (*E*_tot_ in kcal mol^−1^) and ligand efficiencies (kcal mol^−1^Heavy Atom^−1^) for URO A, URO AG, URO B, URO BG, URO C, and URO D ligands computed by molecular docking method.

Binding Site	*E*_tot_ (kcal mol^−1^)					
	URO A	URO AG	URO B	URO BG	URO C	URO D
Sudlow site I	−11.50	−13.59	−11.97	−13.35	−11.57	−11.38
FA9/Cleft	−11.53	−16.03	−11.86	−15.02	−13.14	−12.29
		Ligand efficiencies (kcal mol^−1^Heavy Atom^−1^)				
Sudlow site I	−0.68	−0.68	−0.70	−0.64	−0.64	−0.60
FA9/Cleft	−0.68	−0.80	−0.70	−0.72	−0.73	−0.65

**Table 4:** CHEMPLP total binding energies (*E*_tot_ in kcal mol^−1^) and ligand efficiencies (LE in kcal mol^−1^ Heavy Atom^−1^) for URO A, URO AG, URO B, URO BG, URO C, and URO D ligands docked to FA8 and FA9 binding sites of FA-HSA and heme-HSA.

Binding Site		URO A	URO AG	URO B	URO BG	URO C	URO D
FA-HSA							
FA8	Etot	−14.49	−17.29	−14.14	−18.61	−14.34	−14.29
	LE	−0.85	−0.86	−0.83	−0.89	−0.80	−0.75
FA9/Cleft	Etot	−14.58	−19.08	−13.99	−19.29	−14.47	−15.03
	LE	−0.86	−0.95	−0.82	−0.92	−0.80	−0.79
Hem-HSA							
FA8	Etot	−12.92	−16.87	−13.40	−15.91	−13.46	−12.89
	LE	−0.76	−0.84	−0.79	−0.76	−0.75	−0.68
FA9/Cleft	Etot	−11.23	−16.99	−10.96	−16.24	−11.65	−11.84
	LE	−0.66	−0.85	−0.64	−0.77	−0.65	−0.62

## Data Availability

The datasets generated for this study are available on request to the corresponding author.

## Supplementary Materials

The following supporting information can be downloaded at: https://www.mdpi.com/article/10.3390/molecules29184474/s1, Figure S1. The fluorescence emission spectra of HSA in the presence of an increasing concentration of URO (A) URO A (B) URO AG (C) URO B (D) URO BG (E) URO C (F) URO D at λex = 280 under conditions: pH = 7.4, T = 303 K, respectively. The HSA concentration was 3 × 10^−6^ mol L^−1^, while the URO concentration was increased from 2 × 10^−6^ mol L^−1^ to 10 × 10^−6^ mol L^−1^ at an increment of 1 × 10^−6^ mol L^−1^; Figure S2: The fluorescence emission spectra of HSA in the presence of increasing concentrations of urolithins; Figure S3: Stern–Volmer plots were generated to analyze the quenching of HSA by several urolithins. Figure S4: The impact of the addition of increasing URO concentration on the synchronous fluorescence spectra of HSA at Δλ = 15 nm. Figure S5: Amide I region of FTIR spectra of HSA in the absence and presence of URO (at pH 7.4). Figure S6: Amide II region of FTIR spectra of HSA in the absence and presence of URO (at pH 7.4). Figure S7: Schematic drawings of the interactions of the first GOLD cluster docked solutions of URO A (A), URO AG (B), URO B (C), URO BG (D), URO C (E), and URO D (F) @ Sudlow’s site I of ligand-free HSA (PBD ID 1BM0) generated using LIGPLUS. Dashed lines are hydrogen bonds, and ‘eyelashes’ show residues involved in hydrophobic interactions. Figure S8: Schematic drawings of the interactions of the first GOLD cluster docked solutions of URO A (A), URO AG (B), URO B (C), URO BG (D), URO C (E), and URO D (F) @ FA9/cleft site of ligand-free HSA (PBD ID 1BM0) generated using LIGPLUS. Dashed lines are hydrogen bonds, and ‘eyelashes’ show residues involved in hydrophobic interactions. Figure S9: Schematic drawings of the interactions of the first GOLD cluster docked solutions of URO A (A), URO AG (B), URO B (C), URO BG (D), URO C (E), and URO D (F) @ FA8 site of heme-HSA (PBD ID 1N5U) generated using LIGPLUS. Dashed lines are hydrogen bonds, and ‘eyelashes’ show residues involved in hydrophobic interactions. Figure S10: Schematic drawings of the interactions of the first GOLD cluster docked solutions of URO A (A), URO AG (B), URO B (C), URO BG (D), URO C (E), and URO D (F) @ FA9/cleft site of heme-HSA (PBD ID 1N5U) generated using LIGPLUS. Dashed lines are hydrogen bonds, and ‘eyelashes’ show residues involved in hydrophobic interactions. Figure S11: Schematic drawings of the interactions of the first GOLD cluster docked solutions of URO A (A), URO AG (B), URO B (C), URO BG (D), URO C (E), and URO D (F) @ FA8 site of FA-HSA (PBD ID 8RCP) generated using LIGPLUS. Dashed lines are hydrogen bonds, and ‘eyelashes’ show residues involved in hydrophobic interactions. Figure S12: Schematic drawings of the interactions of the first GOLD cluster docked solutions of URO A (A), URO AG (B), URO B (C), URO BG (D), URO C (E), and URO D (F) @ FA9/cleft site of FA-HSA (PBD ID 8RCP) generated using LIGPLUS. Dashed lines are hydrogen bonds, and ‘eyelashes’ show residues involved in hydrophobic interactions. Figure S13: Cluster analysis of docking results of URO @ Sudlow’s site I (A) and FA9/cleft site (B) of ligand-free HSA (PBD ID 1BM0) using a 1.0 Å RMSD. Figure S14: Cluster analysis of docking results of URO @ FA8 (A) and FA9/cleft site (B) of heme-Fe(III)- and myristic acid-bound HSA HSA (PBD ID 1N5U) using a 1.0 Å RMSD. Figure S15: Cluster analysis of docking results of URO @ FA8 (A) and FA9/cleft site (B) of myristic acid-bound HSA (PBD ID 8RCP) using a 1.0 Å RMSD.
