# Supplementary material for: A Multi-Spectroscopic and Molecular Docking Analysis of the Biophysical Interaction between Food Polyphenols, Urolithins, and Human Serum Albumin
Source: Molecules. 2024 Sep 20;29(18):4474. doi: 10.3390/molecules29184474 (PMC11434395; doi:10.3390/molecules29184474)
Supplement: Supplementary file 1 [file molecules-29-04474-s001.zip › molecules-3188650-supplementary.pdf]

Supplementary material for the article:

A multi-spectroscopic and molecular docking analysis of the biophysical interaction between food polyphenols, urolithins, and human serum albumin

Nevena Zelenović[1], Predrag Ristić[2], Natalija Polović[2], Tamara Todorović[2], Milica Kojadinović[3], Milica Popović[2*]

[1] University of Belgrade-Institute of Chemistry, Technology and Metallurgy, National Institute of the Republic of Serbia, Njegoševa 12, 11000, Belgrade, Serbia;

[2] University of Belgrade-Faculty of Chemistry, Studentski trg 12-16, 11000, Belgrade, Serbia;

[3] University of Belgrade-Institute of Medical Research, National Institute of the Republic of Serbia, Tadeuša Košćuška 1, 11000, Belgrade, Serbia

* Correspondence: la_bioquimica@chem.bg.ac.rs;

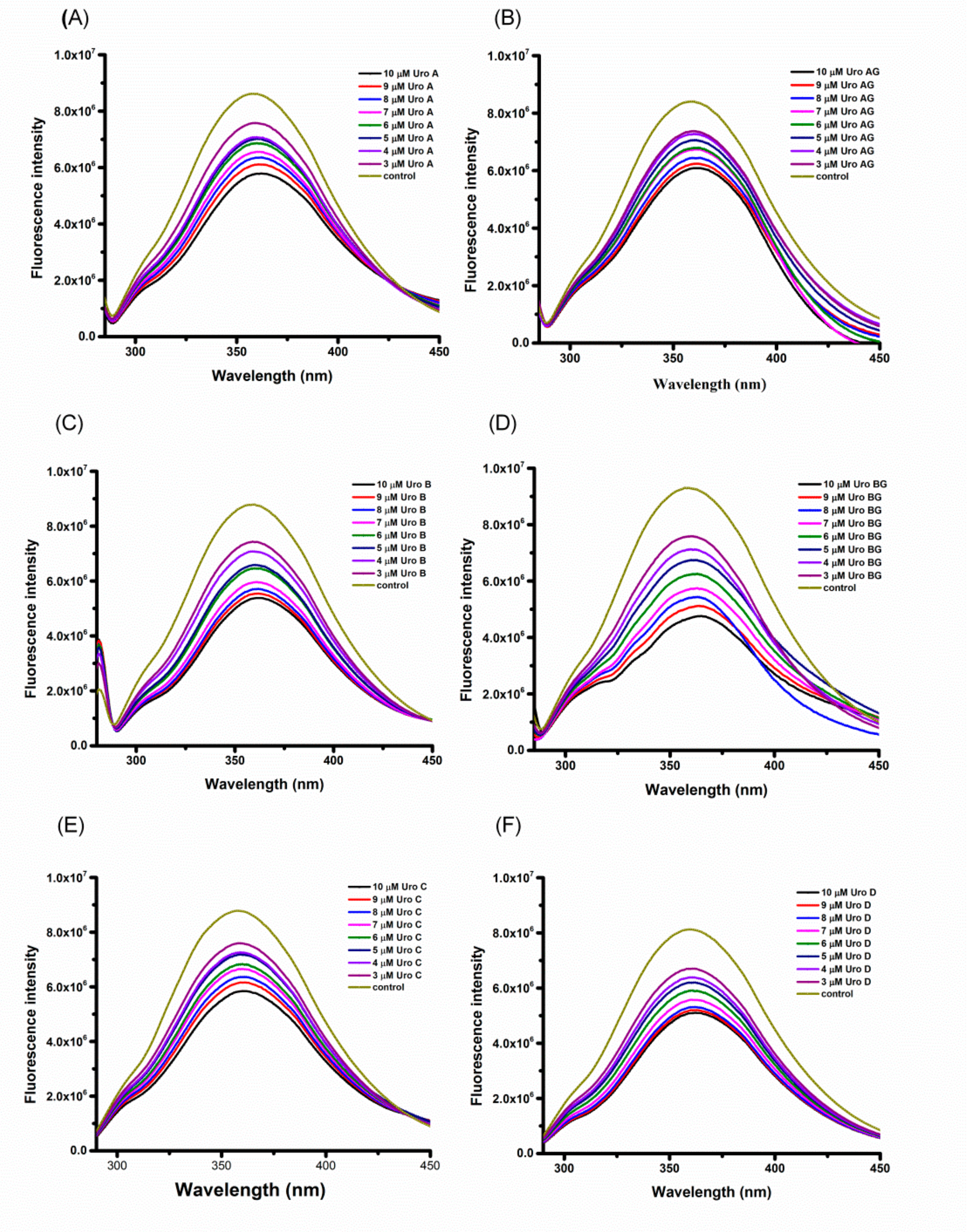

(A)
Fluorescence intensity
Wavelength (nm)
10 μM Uro A
9 μM Uro A
8 μM Uro A
7 μM Uro A
6 μM Uro A
5 μM Uro A
4 μM Uro A
3 μM Uro A
control
(B)
Fluorescence intensity
Wavelength (nm)
10 μM Uro AG
9 μM Uro AG
8 μM Uro AG
7 μM Uro AG
6 μM Uro AG
5 μM Uro AG
4 μM Uro AG
3 μM Uro AG
control
(C)
Fluorescence intensity
Wavelength (nm)
10 μM Uro B
9 μM Uro B
8 μM Uro B
7 μM Uro B
6 μM Uro B
5 μM Uro B
4 μM Uro B
3 μM Uro B
control
(D)
Fluorescence intensity
Wavelength (nm)
10 μM Uro BG
9 μM Uro BG
8 μM Uro BG
7 μM Uro BG
6 μM Uro BG
5 μM Uro BG
4 μM Uro BG
3 μM Uro BG
control
(E)
Fluorescence intensity
Wavelength (nm)
10 μM Uro C
9 μM Uro C
8 μM Uro C
7 μM Uro C
6 μM Uro C
5 μM Uro C
4 μM Uro C
3 μM Uro C
control
(F)
Fluorescence intensity
Wavelength (nm)
10 μM Uro D
9 μM Uro D
8 μM Uro D
7 μM Uro D
6 μM Uro D
5 μM Uro D
4 μM Uro D
3 μM Uro D
control

Figure S1. The fluorescence emission spectra of HSA in the presence of increasing concentration of URO A) URO A B) URO AG C) URO B D) URO BG E) URO C F) URO D at λex = 280 under conditions: pH = 7.4, T = 303 K, respectively. The HSA concentration was 3 × 10^{-6} mol L^{-1} while the URO concentration was increased from 3 × 10^{-6} mol L^{-1} to 10 × 10^{-6} mol L^{-1} at increment of 1 × 10^{-6} mol L^{-1}.

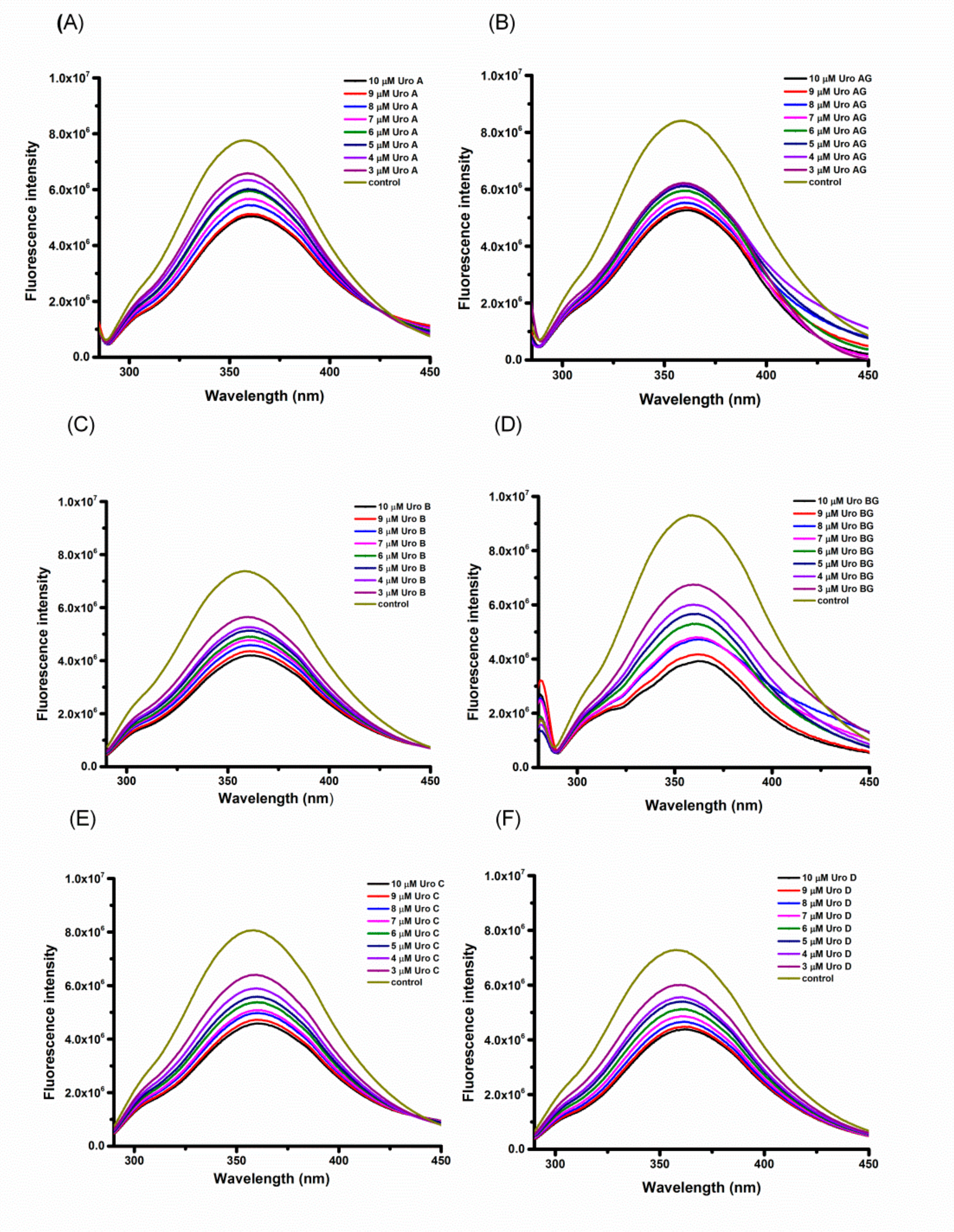
(A)
10 μM Uro A
9 μM Uro A
8 μM Uro A
7 μM Uro A
6 μM Uro A
5 μM Uro A
4 μM Uro A
3 μM Uro A
control
Fluorescence intensity
Wavelength (nm)
(B)
10 μM Uro AG
9 μM Uro AG
8 μM Uro AG
7 μM Uro AG
6 μM Uro AG
5 μM Uro AG
4 μM Uro AG
3 μM Uro AG
control
Fluorescence intensity
Wavelength (nm)
(C)
10 μM Uro B
9 μM Uro B
8 μM Uro B
7 μM Uro B
6 μM Uro B
5 μM Uro B
4 μM Uro B
3 μM Uro B
control
Fluorescence intensity
Wavelength (nm)
(D)
10 μM Uro BG
9 μM Uro BG
8 μM Uro BG
7 μM Uro BG
6 μM Uro BG
5 μM Uro BG
4 μM Uro BG
3 μM Uro BG
control
Fluorescence intensity
Wavelength (nm)
(E)
10 μM Uro C
9 μM Uro C
8 μM Uro C
7 μM Uro C
6 μM Uro C
5 μM Uro C
4 μM Uro C
3 μM Uro C
control
Fluorescence intensity
Wavelength (nm)
(F)
10 μM Uro D
9 μM Uro D
8 μM Uro D
7 μM Uro D
6 μM Uro D
5 μM Uro D
4 μM Uro D
3 μM Uro D
control
Fluorescence intensity
Wavelength (nm)

Figure S2. The fluorescence emission spectra of HSA in the presence of increasing concentration of URO A) URO A B) URO AG C) URO B D) URO BG E) URO C F) URO D at λex = 280 under conditions: pH = 7.4, T = 310 K, respectively. The HSA concentration was 3×10^{-6} mol L^{-1} while the URO concentration was increased from 3×10^{-6} mol L^{-1} to 10×10^{-6} mol L^{-1} at increment of 1×10^{-6} mol L^{-1}.

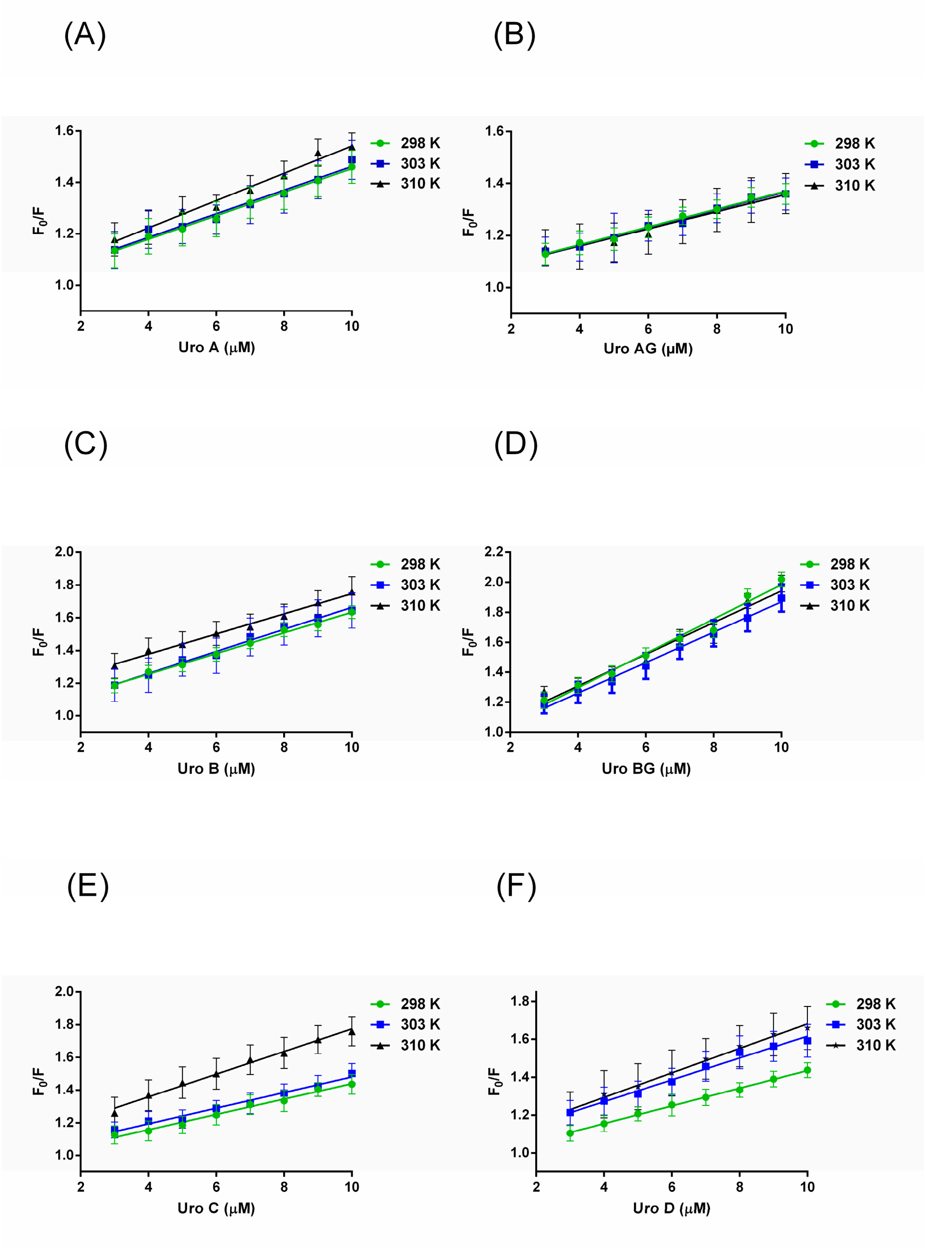

(A)
(B)
(C)
(D)
(E)
(F)
F0/F
298 K
303 K
310 K
Uro A (μM)
Uro AG (μM)
Uro B (μM)
Uro BG (μM)
Uro C (μM)
Uro D (μM)

Figure S3. Stern-Volmer plots were generated to analyze the quenching of HSA by several compounds, namely **A)** URO A **B)** URO AG **C)** URO B **D)** URO BG **E)** URO C **F)** URO D, at temperatures of 298 K, 303 K and 310 K, and a pH value of 7.4. The HSA concentration was 3×10^{-6} mol L^{-1} while the URO concentration was increased from 3×10^{-6} mol L^{-1} to 10×10^{-6} mol L^{-1} at increment of 1×10^{-6} mol L^{-1}. Error bars indicate standard errors of triplicate measurements

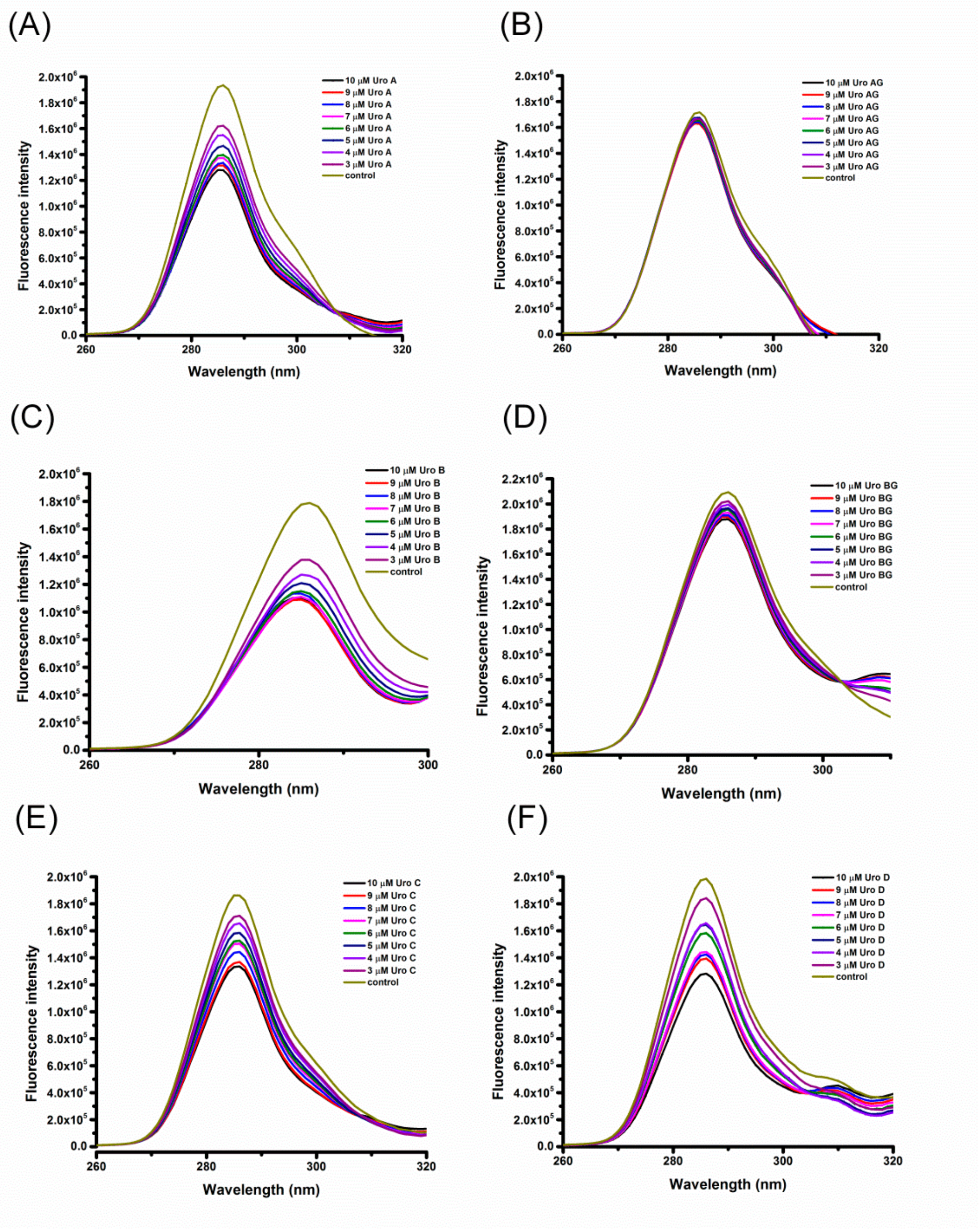
(A)
10 μM Uro A
9 μM Uro A
8 μM Uro A
7 μM Uro A
6 μM Uro A
5 μM Uro A
4 μM Uro A
3 μM Uro A
control
Fluorescence intensity
Wavelength (nm)
(B)
10 μM Uro AG
9 μM Uro AG
8 μM Uro AG
7 μM Uro AG
6 μM Uro AG
5 μM Uro AG
4 μM Uro AG
3 μM Uro AG
control
Fluorescence intensity
Wavelength (nm)
(C)
10 μM Uro B
9 μM Uro B
8 μM Uro B
7 μM Uro B
6 μM Uro B
5 μM Uro B
4 μM Uro B
3 μM Uro B
control
Fluorescence intensity
Wavelength (nm)
(D)
10 μM Uro BG
9 μM Uro BG
8 μM Uro BG
7 μM Uro BG
6 μM Uro BG
5 μM Uro BG
4 μM Uro BG
3 μM Uro BG
control
Fluorescence intensity
Wavelength (nm)
(E)
10 μM Uro C
9 μM Uro C
8 μM Uro C
7 μM Uro C
6 μM Uro C
5 μM Uro C
4 μM Uro C
3 μM Uro C
control
Fluorescence intensity
Wavelength (nm)
(F)
10 μM Uro D
9 μM Uro D
8 μM Uro D
7 μM Uro D
6 μM Uro D
5 μM Uro D
4 μM Uro D
3 μM Uro D
control
Fluorescence intensity
Wavelength (nm)

Figure S4. The impact of addition of increasing URO concentration on the synchronous fluorescence spectra of HSA at $\Delta\lambda = 15$ nm: A) URO A; B) URO AG; C) URO B; D) URO BG; E) URO C; F) URO D. The HSA concentration was 3×10^{-6} mol L^{-1}, while the URO concentrations ranged 3×10^{-6} - 10×10^{-6} mol L^{-1} from top to bottom.

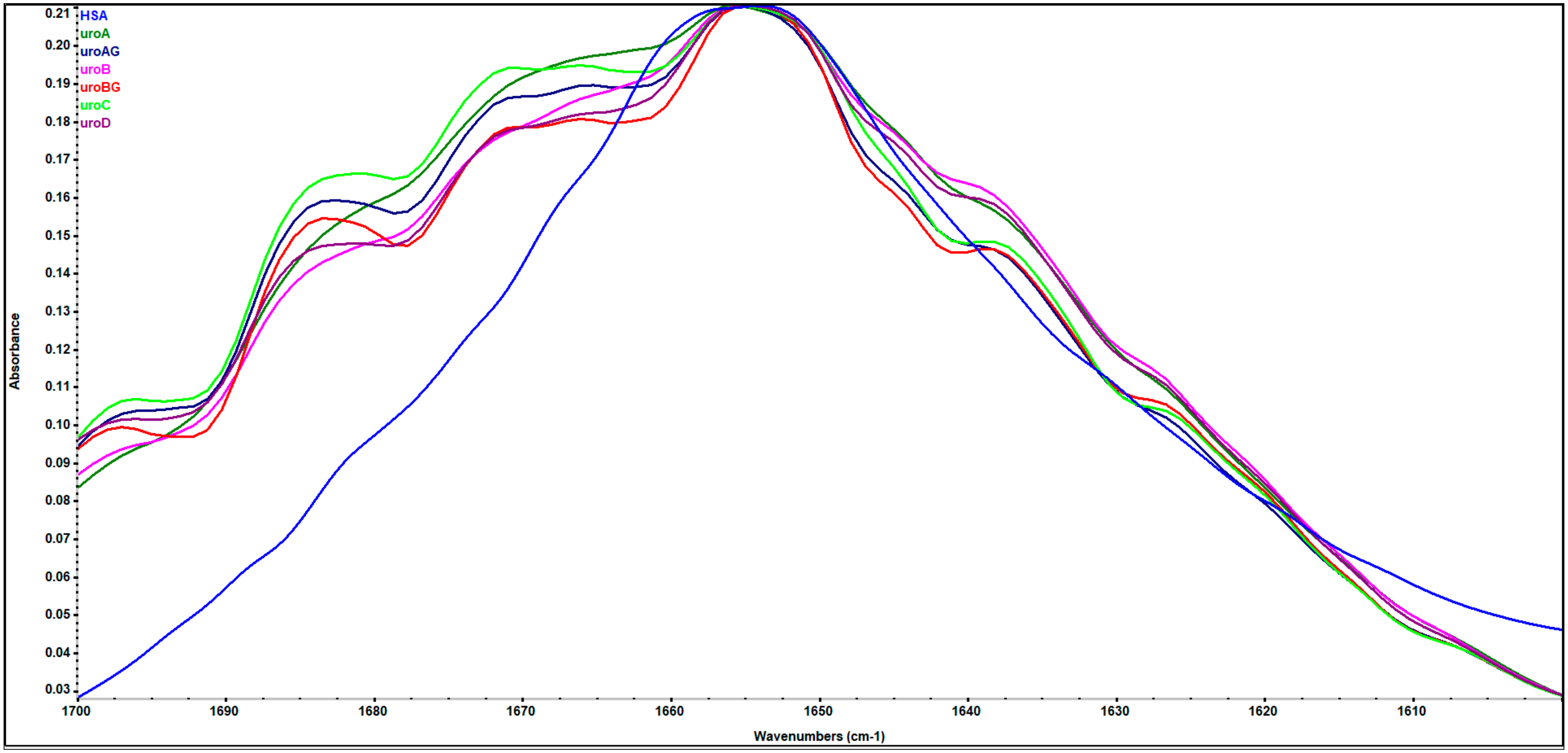

Figure S5. Amide I region of FTIR spectra of HSA in the absence and presence of URO (at pH 7.4)

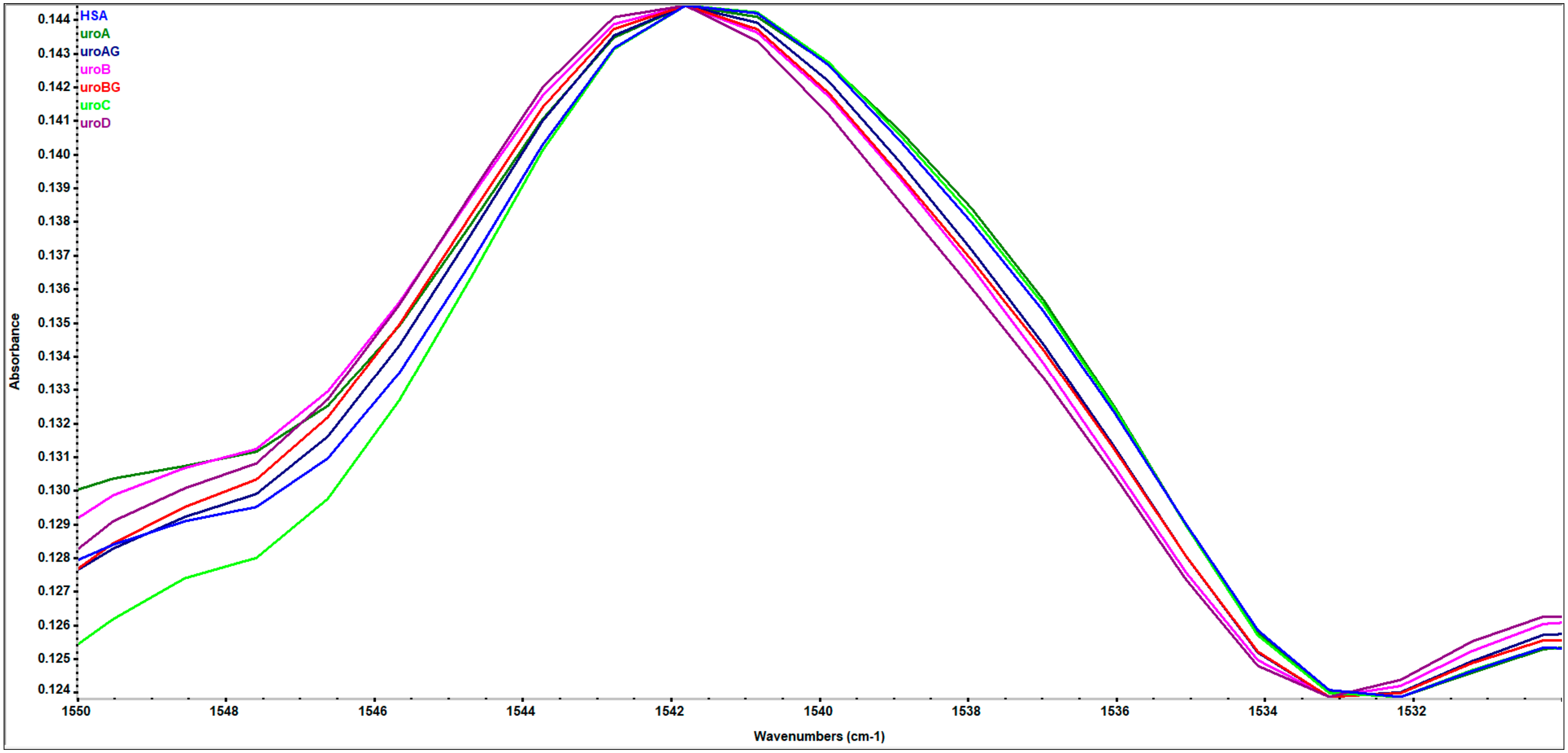

Figure S6. Amide II region of FTIR spectra of HSA in the absence and presence of URO (at pH 7.4).

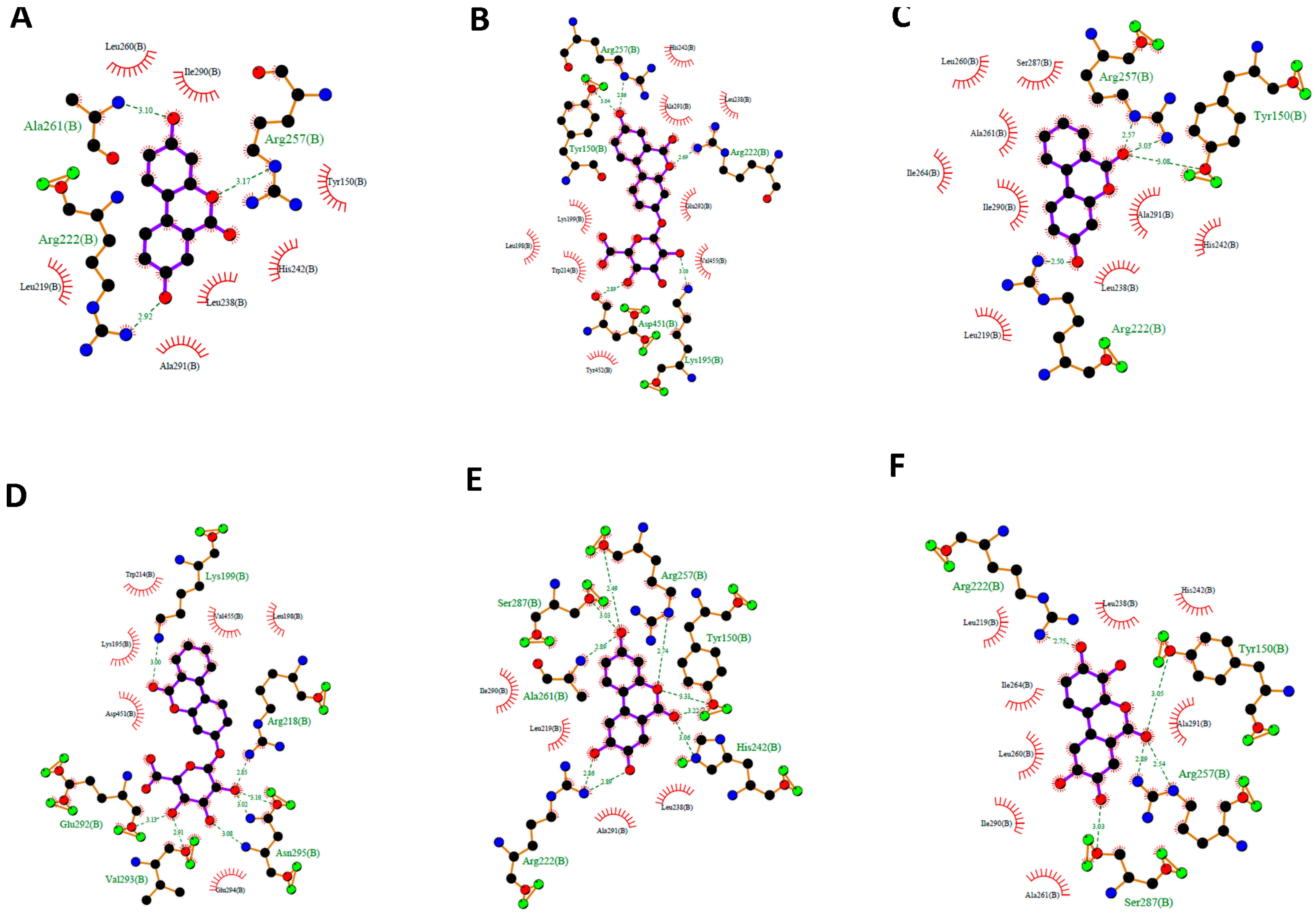

Figure S7. Schematic drawings of the interactions of the first GOLD cluster docked solutions of URO A (A), URO AG (B), URO B (C), URO BG (D), URO C (E) and URO D (F) @ Sudlow's site I of ligand-free HSA (PBD ID 1BM0) generated using LIGPLUS. Dashed lines are hydrogen bonds and 'eyelashes' show residues involved in hydrophobic interactions.

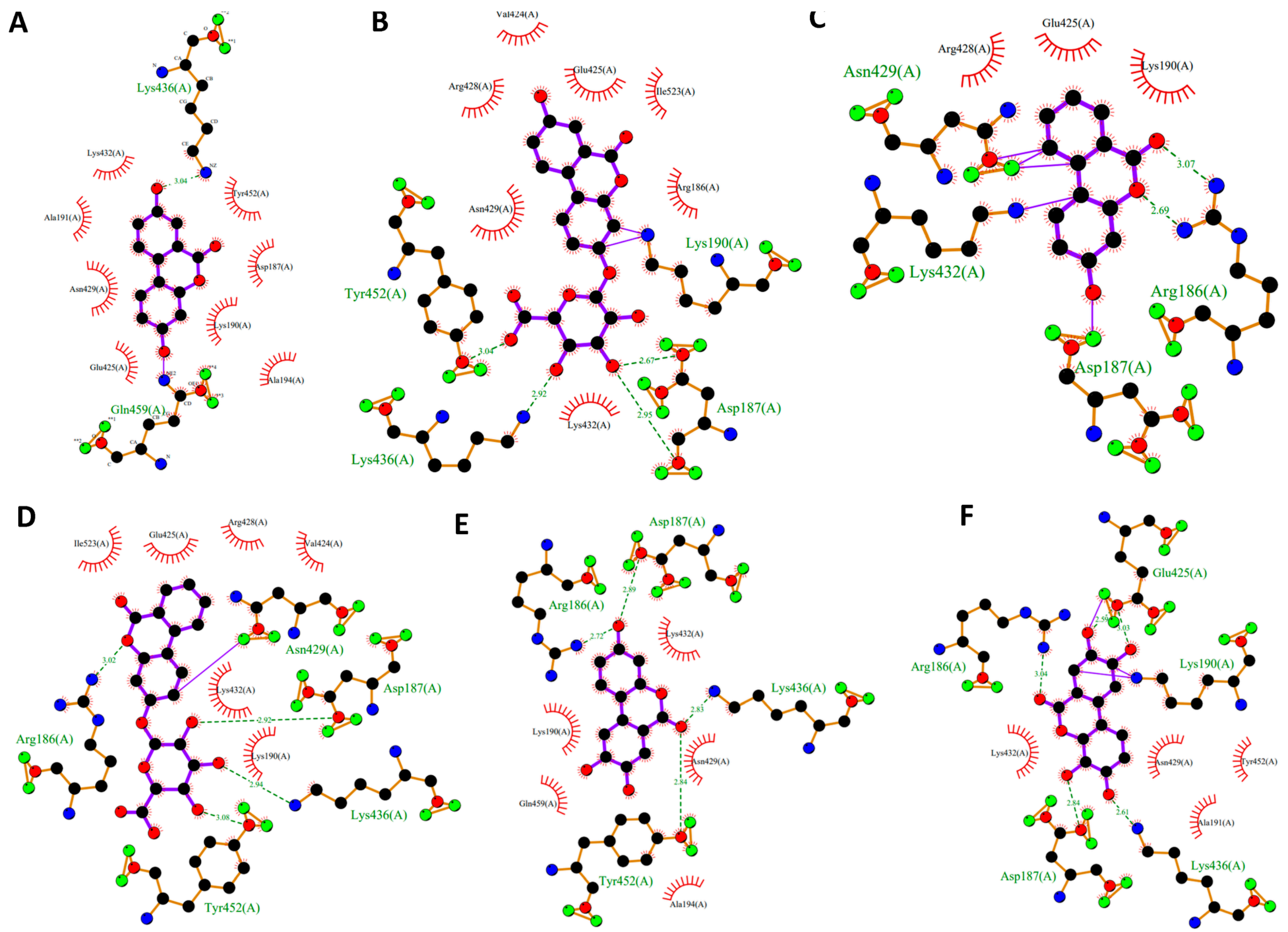

Figure S8. Schematic drawings of the interactions of the first GOLD cluster docked solutions of URO A (A), URO AG (B), URO B (C), URO BG (D), URO C (E) and URO D (F) @ FA9/cleft site of ligand-free HSA (PBD ID 1BM0) generated using LIGPLUS. Dashed lines are hydrogen bonds and ‘eyelashes’ show residues involved in hydrophobic interactions.

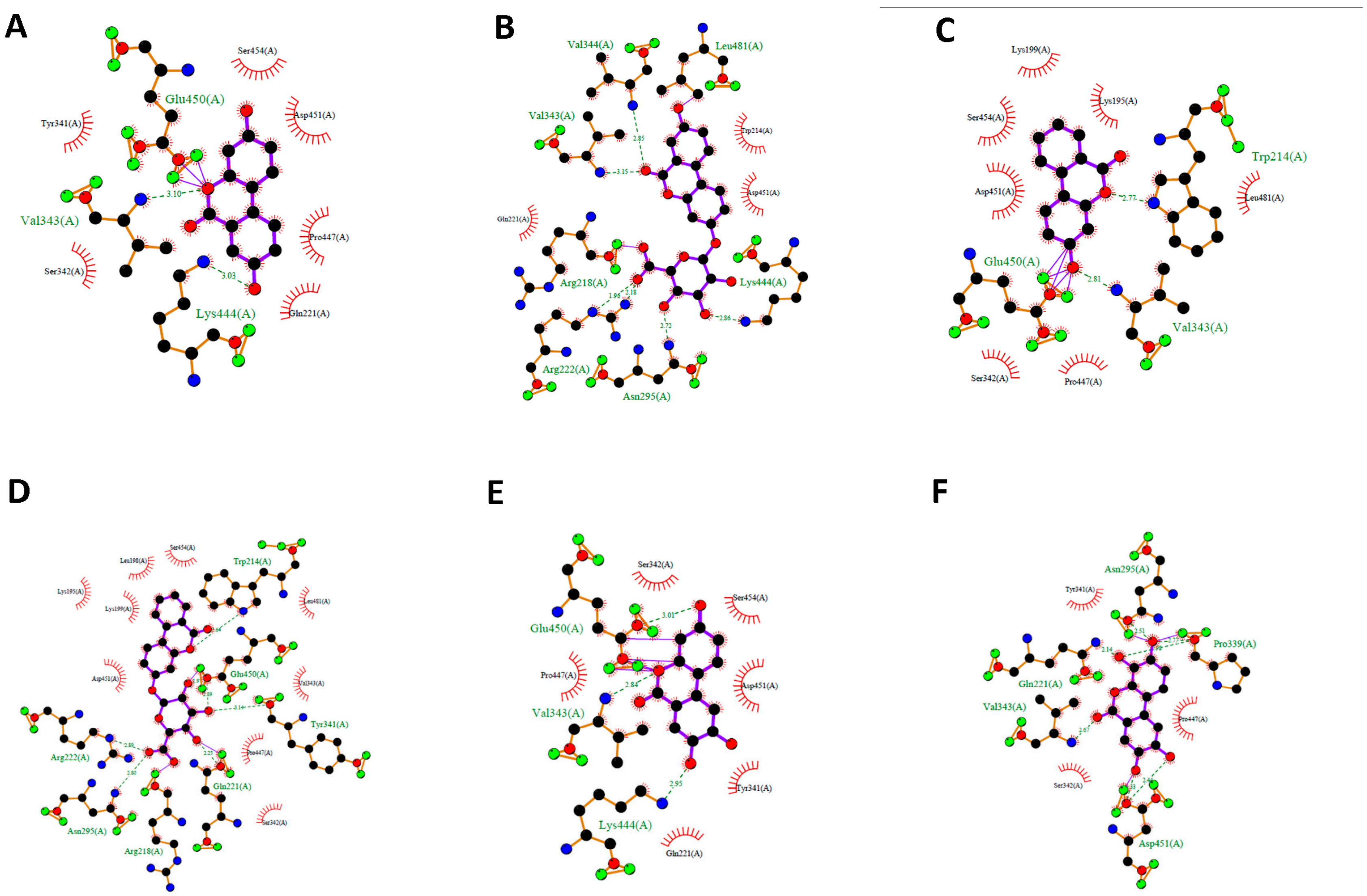

Figure S9. Schematic drawings of the interactions of the first GOLD cluster docked solutions of URO A (A), URO AG (B), URO B (C), URO BG (D), URO C (E) and URO D (F) @ FA8 site of heme-HSA (PBD ID 1N5U) generated using LIGPLUS. Dashed lines are hydrogen bonds and ‘eyelashes’ show residues involved in hydrophobic interactions.

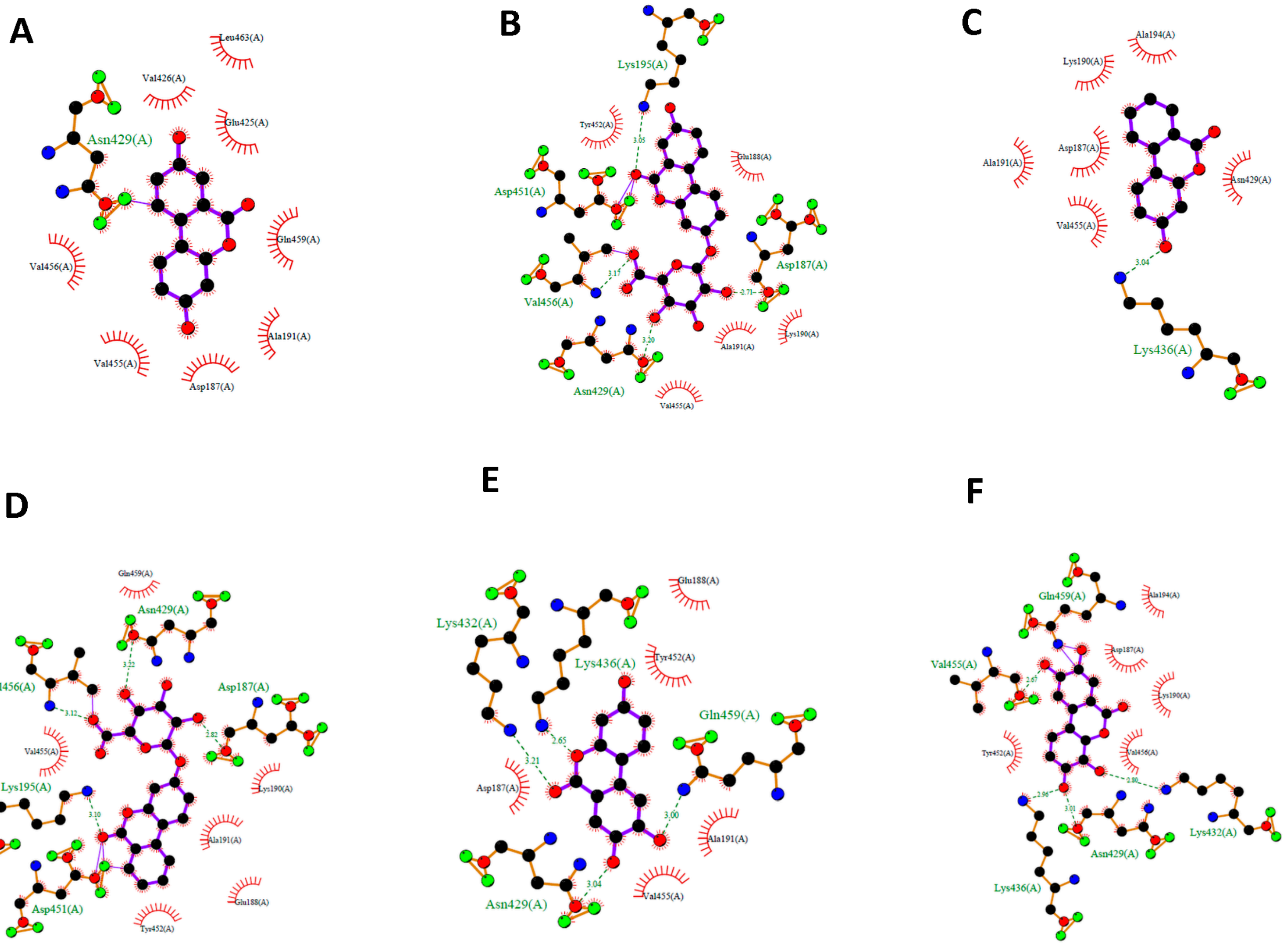

Figure S10. Schematic drawings of the interactions of the first GOLD cluster docked solutions of URO A (A), URO AG (B), URO B (C), URO BG (D), URO C (E) and URO D (F) @ FA9/cleft site of heme-HSA (PBD ID 1N5U) generated using LIGPLUS. Dashed lines are hydrogen bonds and ‘eyelashes’ show residues involved in hydrophobic interactions.

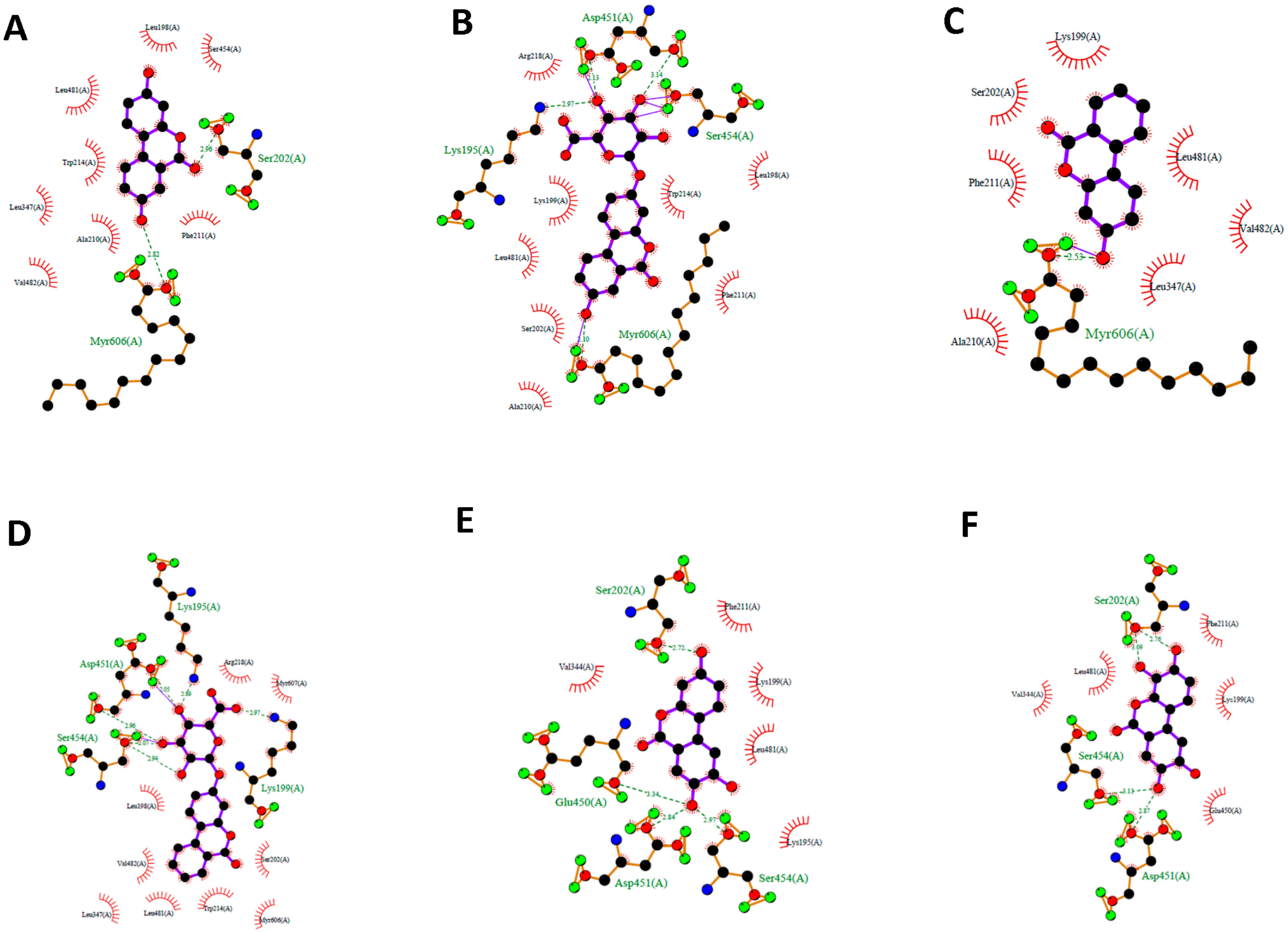

Figure S11. Schematic drawings of the interactions of the first GOLD cluster docked solutions of URO A (A), URO AG (B), URO B (C), URO BG (D), URO C (E) and URO D (F) @ FA8 site of FA-HSA (PBD ID 8RCP) generated using LIGPLUS. Dashed lines are hydrogen bonds and 'eyelashes' show residues involved in hydrophobic interactions.

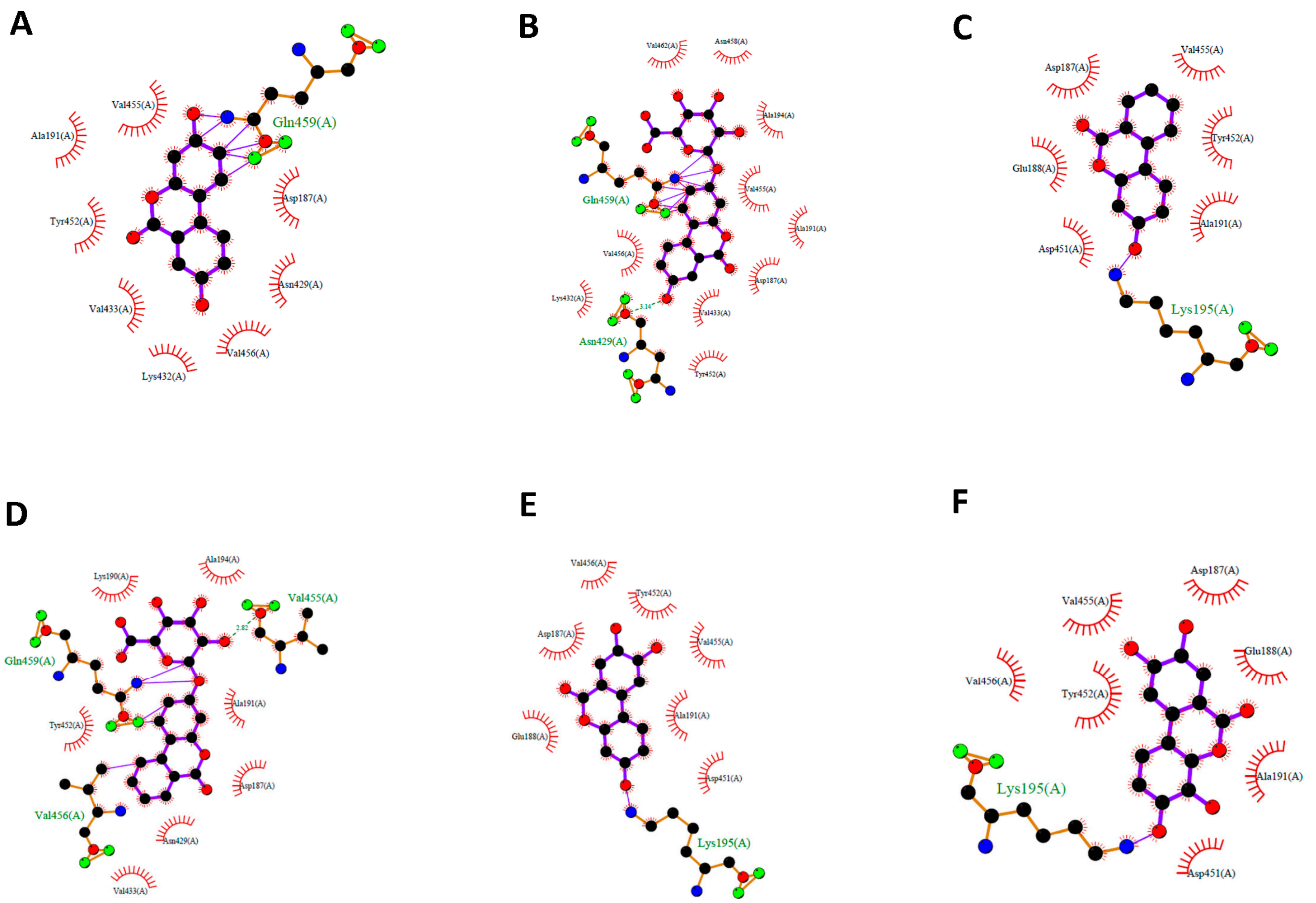

Figure S12. Schematic drawings of the interactions of the first GOLD cluster docked solutions of URO A (A), URO AG (B), URO B (C), URO BG (D), URO C (E) and URO D (F) @ FA9/cleft site of FA-HSA (PBD ID 8RCP) generated using LIGPLUS. Dashed lines are hydrogen bonds and 'eyelashes' show residues involved in hydrophobic interactions.

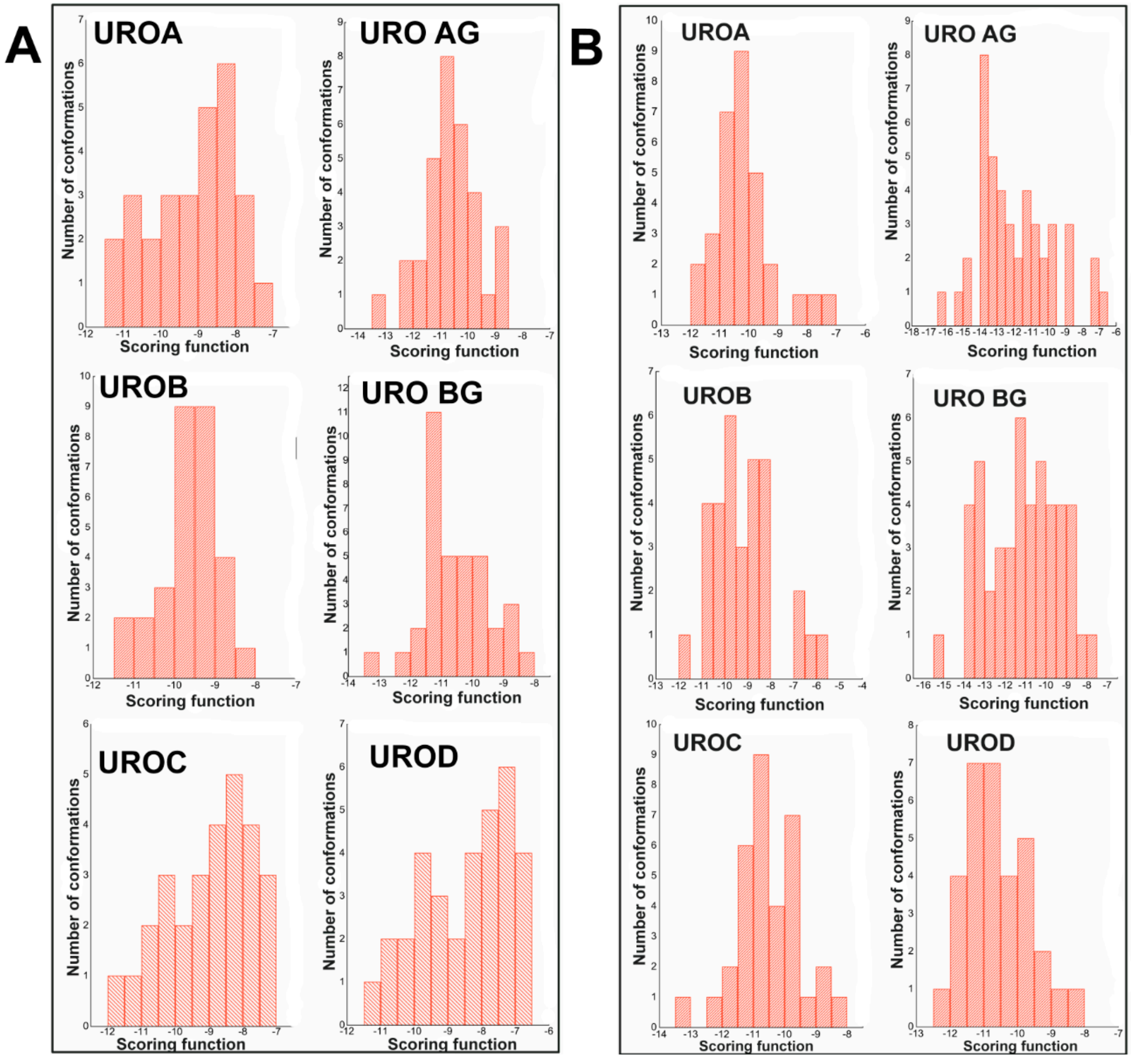

Figure S13. Cluster analysis of docking results of URO @ Sudlow's site I (A) and FA9/cleft site (B) of ligand-free HSA (PBD ID 1BM0) using a 1.0 Å RMSD.

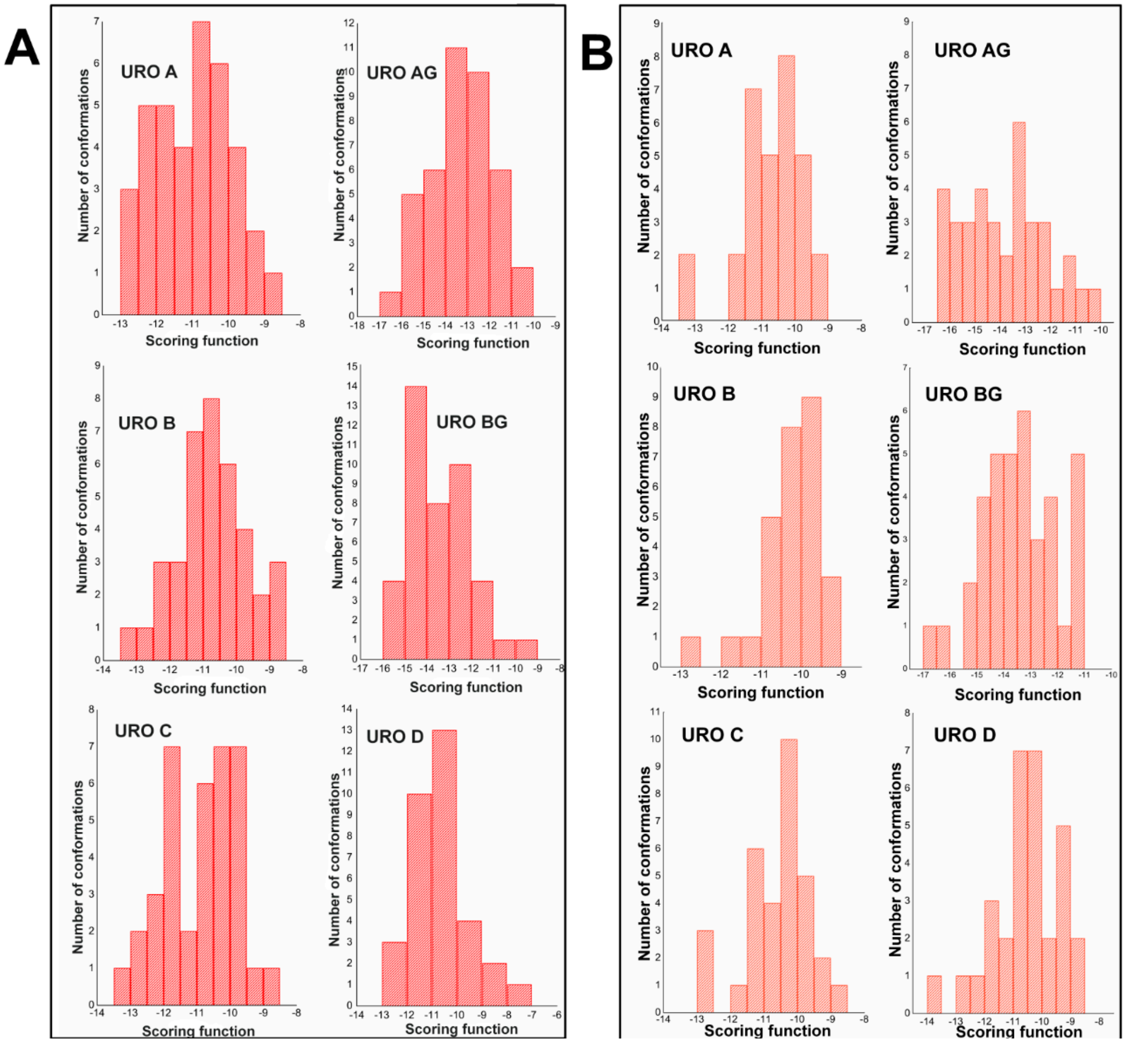

Figure S14. Cluster analysis of docking results of URO @ FA8 (A) and FA9/cleft site (B) of heme-Fe(III)- and myristic acid-bound HSA HSA (PBD ID 1N5U) using a 1.0 Å RMSD.

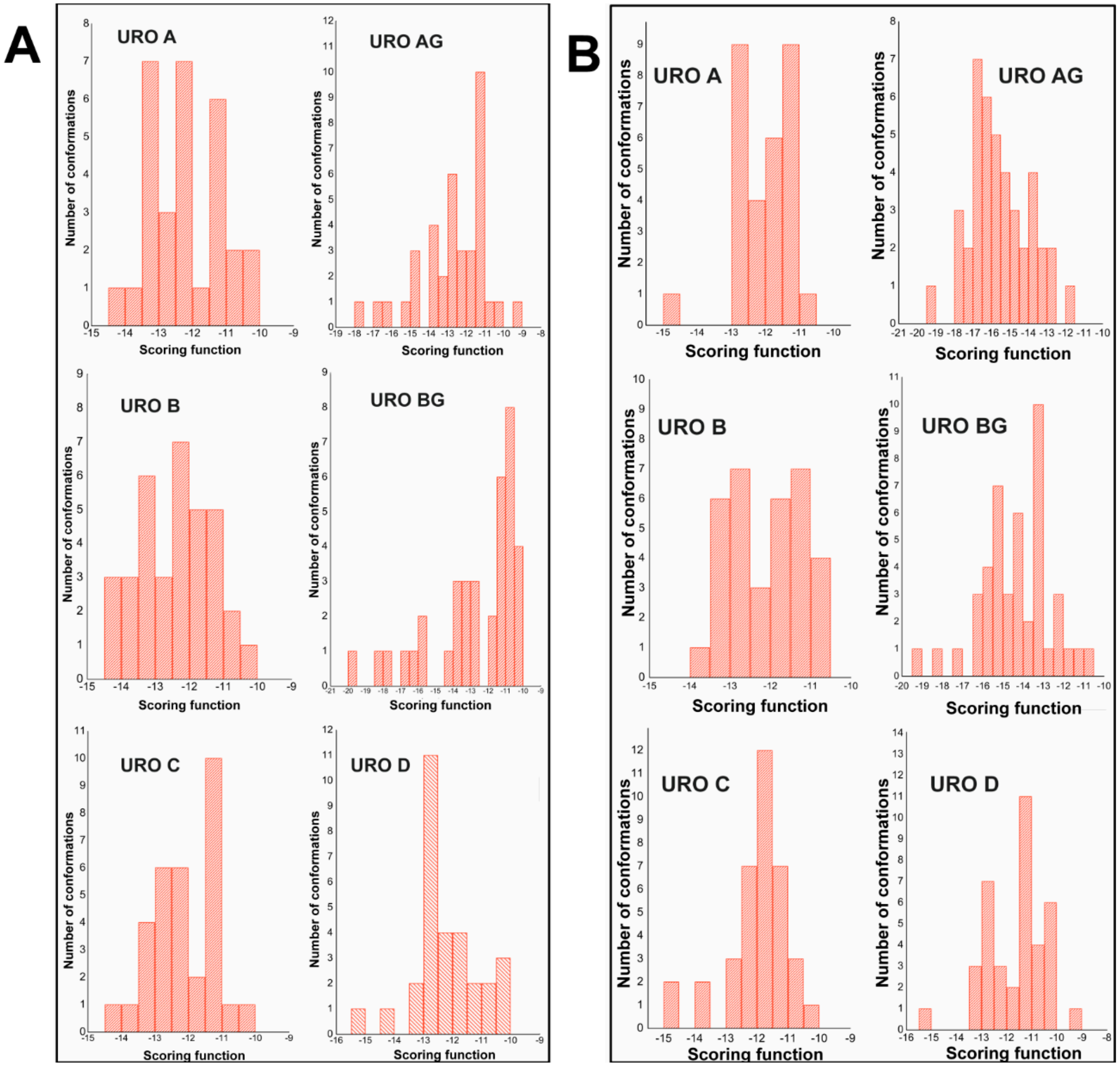

Figure S15. Cluster analysis of docking results of URO @ FA8 (A) and FA9/cleft site (B) of myristic acid-bound HSA (PBD ID 8RCP) using a 1.0 Å RMSD.
